# A semi-dominant mutation in a CC-NB-LRR-type protein leads to a short-root phenotype in rice

**DOI:** 10.1186/s12284-018-0250-1

**Published:** 2018-10-03

**Authors:** Zhiming Yu, Lixiang Dong, Zhifang Jiang, Keke Yi, Jianhua Zhang, Zhongchen Zhang, Zhenxing Zhu, Yuhuan Wu, Maojun Xu, Jun Ni

**Affiliations:** 1 College of Life and Environmental Science, Hangzhou Normal University, No. 16 Xuelin Street, Hangzhou, 310018 Xiasha District China; 2 Key Laboratory of Plant Nutrition and Fertilizers, Ministry of Agriculture, Institute of Agricultural Resources and Regional Planning, Chinese Academy of Agricultural Sciences, Beijing, 10081 China; 3 Faculty of Science, Hong Kong Baptist University, Hong Kong, China; 4 College of Agriculture, Northeast Agricultural University, Harbin, 150030 China; 5 Agricultural Crops Molecular Improving Lab, Liaoning Academy of Agricultural Sciences, Shenyang, 110161 China

**Keywords:** Defense, Mutation, Necrotic, NO, Pathogen, Rice, Root, ROS

## Abstract

The mechanisms of plant defense against pathogen attack in plant leaves have been extensively studied. However, our understanding of plant defense mechanisms in plant roots is still limited. In this study, a semi-dominant mutant *nrtp1-D* (*necrotic root tip 1*), with a short-root phenotype, was characterized in rice. Map-based cloning revealed that *NRTP1* encoded a typical coiled-coil nucleotide binding leucine rich repeat (CC-NB-LRR) type protein and the mutation caused an amino acid substitution in the Nucleotide-Binding adaptor shared by Apaf1, certain R genes and CED4 (NB-ARC) domain, which may cause constitutive auto-activation of the NRTP1 protein. Gene expression analysis revealed that *NRTP1* was preferentially expressed in rice roots. Expression of mutant *nrtp1-D* in tobacco leaves induced necrotic lesions, which indicated a common mechanism of plant defense response between leaves and roots. Transcriptome analysis revealed that many typical defense-response genes were differentially expressed in homozygous *nrtp1-D*. In addition, we also found differential expression of genes in pathways which had not previously been described as being associated with pathogen response. Histochemical analysis showed that the level of nitric oxide (NO), but not reactive oxygen species (ROS), was increased in homozygous *nrtp1-D* mutant roots. These results indicate that, in addition to the mechanism of defense response common to both roots and shoots, a novel pathway may also exist in rice roots, which does not operate in shoots.

**Electronic supplementary material:**

The online version of this article (10.1186/s12284-018-0250-1) contains supplementary material, which is available to authorized users.

## Background

Rice (*Oryza sativa* L.) is not only a model plant for monocotyledonous species, but is also the most important staple food, feeding half of the world’s population. Rice diseases are among the major constraints to sustainable rice production (Dai et al. 2007). Thus, studies of rice defense responses are of great interest for both advancing our mechanistic knowledge of plant-pathogen interaction and for accelerating crop improvement (Chen and Ronald 2011).

When plants are invaded by pathogens, pathogen-associated molecular patterns (PAMPs) are recognized and a basal resistance response, called PAMP-triggered immunity (PTI), is triggered in plants. To overcome the defense responses caused by PTI, some pathogens secrete effectors to increase pathogen virulence. Plants, in turn, employ resistance (R) proteins to interact with these effectors to induce a resistance response called effector-triggered immunity (ETI). Typically, a localized programmed cell death, the hypersensitive response (HR), is associated with ETI to restrict pathogen growth in plant cells (Dangl et al. 2013; Jones and Dangl 2006).

The largest family of R proteins is the nucleotide-binding site leucine-rich repeat (NB-LRR) family of proteins (Tameling and Takken 2008). This large family is encoded by hundreds of genes per plant genome, and can be subdivided into two subfamilies: Toll/interleukin-1 receptor (TIR)-NB-LRRs (TNLs) and coiled-coil (CC)-NB-LRRs (CNLs) (McHale et al. 2006). The N-terminal TIR or CC domains are involved in the formation of dimers to activate defense signaling. The central NB-ARC domain (the nucleotide-binding adaptor shared by Apaf1, certain R gene products and CED4) acts as a nucleotide-binding pocket and hydrolyzes ATP to induce conformational changes in NB-LRR proteins, and plays important roles in controlling protein activity. The C-terminal LRR domain interacts with the NB-ARC domain to keep the NB-LRR protein in an auto-inhibited state in the absence of pathogen effectors. Upon pathogen attack, the LRR domain interacts with effectors, releasing the auto-inhibition state of NB-LRR to activate defense signaling (Takken and Goverse 2012). Disruption of the interaction between the LRR domain and the NB-ARC domain results in constitutive activation of plant disease responses, coupled with programmed cell death (PCD), in the absence of pathogen attack (Ade et al. 2007; Bendahmane et al. 2002; Michael Weaver et al. 2006; Rairdan and Moffett 2006). Specifically, amino acid substitutions in the NB-ARC domain of NLS1, a CC-NB-LRR-type R protein in rice, caused constitutive auto-activation of the NLS1 protein, resulting in spontaneous lesions in rice leaves (Tang et al. 2011).

In addition to the roles of plant defense hormones (Robert-Seilaniantz et al. 2011), nitric oxide (NO) and reactive oxygen species (ROS) are also important signaling molecules in plant defense response to pathogen attack (Scheler et al. 2013). Upon pathogen recognition, both NO and ROS function as signals in plant defense responses (Wang et al. 2013). Non-expresser of pathogen-related gene 1 (NPR1), a key player controlling defense gene expression, is affected by both NO and ROS regulation (Lindermayr et al. 2010; Peleg-Grossman et al. 2010). Furthermore, NO and ROS are tightly regulated by one another via a complex mechanism. For example, ROS production is inhibited by S-nitrosylation, while NO production is also required for the induction of ROS accumulation (Rasul et al. 2012; Yun et al. 2011). This apparent contradiction indicates a complex relationship between NO and ROS signaling in plant defense responses. Accumulation of ROS was observed in *nls1-D* mutant leaves, which was considered to be part of defense responses in rice leaves (Tang et al. 2011).

In the current research, we describe the isolation and characterization of a semi-dominant rice mutant, *nrtp1-D* (*necrotic root tip 1*), which displayed short roots and defense-related phenotypes. Map-based cloning revealed an amino acid substitution in the NB-ARC domain of a typical CC-NB-LRR-type protein. Our results indicated that, in addition to the mechanism of defense response common to both roots and shoots, a novel pathway may also exist in rice roots, which is not expressed in shoots.

## Methods

### Plant materials and growth conditions

The rice (*Oryza sativa*) seedlings were grown in culture solution (Yoshida et al. 1976) in a growth chamber with 12 h of light at 28 °C and 12 h of dark at 22 °C. The light intensity was 4000 lx and the humidity was 70%. The root and shoot lengths of 7-d-old rice seedlings were measured for comparison. For the resin sectional analysis of roots, primary root tips were selected and the procedures of staining, dehydration, cleaning, infiltration and embedding were performed as previously described (Liu et al. 2005).The rice mutant was isolated from an EMS-mutagenized M_2_ population derived from the *indica* cultivar Kasalath, and the *nrtp1-D* heterozygous mutant was initially identified for its short root phenotype.

### Map-based cloning of *NRTP1*

For the positional cloning of the mutated gene, an *nrtp1-D* heterozygous mutant was crossed with *O. sativa* ssp. *japonica* Nipponbare wild type, to generate an F_2_ mapping population. The map-based cloning was carried out as previously described (Ni et al. 2011). To confirm the result of map-based cloning, a derived cleaved amplified polymorphic sequences (dCAPS) marker was developed and the PCR products were digested using *Eco*91I (Thermo Fisher Scientific). Markers mentioned in this experiment are listed in Additional file 1: Table S1.

### Complementation of *nrtp1-D* mutant

For the reverse complementation test, an 8 kb genomic fragment, containing the entire *nrtp1-D* gene (including two exons and one intron) and 2.7 kb upstream and 1.6 kb downstream sequences, was amplified from the genome of the homozygous *nrtp1-D* mutant using PCR and the primers HFBP-U and HFBP-L. The DNA fragment was first cloned into the *pDONR201* entry vector, and then shuttled into the Gateway destination vector *pGWB501* as previously described (Ni et al. 2016). The vector was transformed into ‘Kasalath’ wild type by the *Agrobacterium*-mediated method (Ni et al. 2014a). We generated five independent lines and the phenotype of T_2_ generation transgenic rice was compared with that of the wild type. We chose two independent lines for complementation test. Primers used in this experiment are listed in Additional file 1: Table S1.

### RNA isolation and RT-PCR analysis

For tissue expression studies, total RNA was extracted from different tissues of Kasalath wild type including shoots, stem bases and roots of 7-d-old seedlings, and the stem and young panicle of adult plants. For *NRTP1* expression analysis after hormone treatments, 7-d-old wild-type seedlings were transferred to 1 mM salicylic acid (SA) or jasmonic acid (JA) solution for 6 h. Meanwhile, leaves were sprayed with SA and JA solution with 0.01% Tween 20. Untreated roots were used as a positive control. For characterization of *35Sp:NRTP1* transgenic rice, RNA from leaves of 7-d-old seedlings was extracted. The expression of *NRTP1* was analyzed by RT-PCR, using primers 410RT-U and 410RT-L. The expression level of *OsActin* was used as an internal control. Primers used in these experiments are listed in Additional file 1: Table S1.

### Transcriptome analysis

Total RNA was extracted from the whole plant of 7-d-old Kasalath wild type and homozygous *nrtp1-D* mutant seedlings. Three biological replicates were set up. The RNA library construction and sequencing were performed as previously described (Ni et al. 2018). Illumina Hiseq2500 was used in the paired-end sequencing. The information of basic sequencing data is listed in Additional file 2: Table S2. The reads were aligned to the rice reference genome (http://genome.jgi.doe.gov) using Tophat package. The aligned read files were processed by Cufflinks, which uses the normalized sequence fragment counts to measure the relative abundances of the transcripts. Only those comparisons with q values less than 0.01 and status marked as “OK” in the Cuffdiff output were regarded as differentially expressed genes. Kyoto Encyclopedia of Genes and Genomes (KEGG) and Gene Ontology (GO) analyses were performed as previously described (Ni et al. 2018).

### Construction and characterization of transgenic plants

For the construction of *NRTP1p:GUS* transgenic rice, a 2.2 kb promoter fragment was amplified by Pro-U and Pro-L. The PCR product was digested by *Kpn* I and *Sal* I, and then cloned into the binary vector *pCAMBIA1300NH-plus GUS* (Qin et al. 2013). The T_2_ generation of three independent transgenic lines was used in this experiment. The histochemical GUS analysis was performed as previously described (Jefferson et al. 1987). Leaves and flowers were collected form mature plants and the rest of the tissues were collected from the 7-d-old seedlings. The transverse sections of roots were carried out using a vibratome (DTK-1000), and longitudinal and transverse sections at the shoot-root junctions were carried out manually.

For the construction of *35Sp:NRTP1* and *35Sp:nrtp1-D* transgenic rice, full length cDNA of *NRTP1* and *nrtp1-D* was amplified by PCR using Over-U and Over-L. The PCR product was digested by *BamH* I and *Sal* I, and then cloned into the binary vector *35S-pCAMBIA1301*, which had the *35S* promoter to drive these coding sequences (Zhang et al. 2015). In addition to screening for resistance, positive lines were also characterized by checking the expression of *NRTP1* in leaves, using RT-PCR. *NRTP1* was not expressed in wild-type rice leaves, so the leaves expressing *NRPT1* would be transgenic positive lines. We obtained eight independent lines of *35Sp:NRTP1* transgenic rice, and the T_2_ generation of two lines was used in this experiment.

*Agrobacterium*-mediated transient transformation of *Nicotiana benthamiana* was performed as previously described (Ni et al. 2016). Leaves of 4-week-old plants were infiltrated with bacterial cultures and observed for necrotic lesions every 12 h for 96 h. Constructions used for the transgenic rice were also used in tobacco experiment (*35Sp:NRTP1*, *35Sp:nrtp1-D* and the empty vector *35S-pCAMBIA1301*). Three different controls were used in this experiment. They were water control, empty vector control and *35Sp:NRTP1* control. Primers used in these experiments are listed in Additional file 1: Table S1.

### Histochemistry in root tip

The primary root tips of 7-d-old seedlings from wild type and homozygous *nrtp1-D* were used to perform histochemistry experiments. Cell death detection was observed by Evans blue staining, performed as previously described (Qin et al., 2013). The endogenous NO level was detected by DAF-FM DA as previously described (Zhao et al. 2009). For the histochemical detection of H_2_O_2_ and O_2_^.-^ (NBT) and ROS (HPF), staining was performed as previously described (Xu et al. 2017). For positive control of HPF staining, wild-type root tips were submerged with 0.1% H_2_O_2_ for 10 min before staining. For the quantification of NO and H_2_O_2_ contents, leaves and root tips (~ 1 cm) of 7-d-old seedlings were collected. NO and H_2_O_2_ contents were measured using the nitric oxide assay kit (Beyotime) and hydrogen peroxide assay kit (Beyotime) following the vendor’s instructions.

## Results

### Isolation and characterization of *nrtp1-D*

**Fig. 1:**
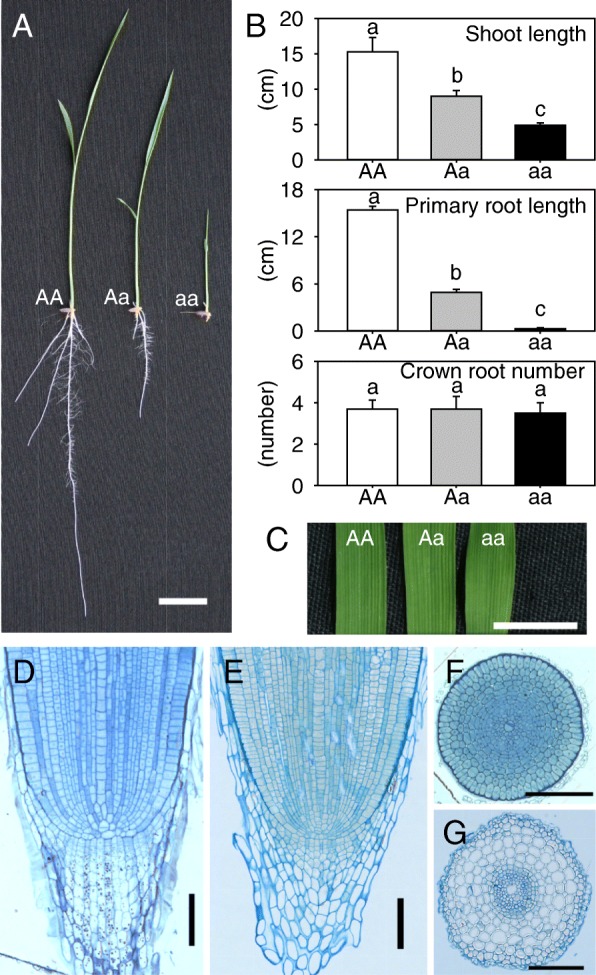
Phenotypes of 7-d-old *nrtp1-D* mutant seedlings. (**a**) Overall comparison of phenotypes between wild type and *nrtp1-D* mutant seedlings. AA, wild-type ‘Kasalath’ plants; Aa, *nrtp1-D* heterozygous plants; aa, *nrtp1-D* homozygous plants. Bar = 2 cm. (**b**) Shoot length, primary root length and crown root number of *nrtp1-D* mutant seedlings. Samples with different letters are significantly different (*P* < 0.01; Tukey’s test). Values are mean ± SD of three biological replicates. White, AA; grey, Aa; black, aa. (**c**) Comparison of leaves among AA, Aa and aa. Bar = 0.5 cm. (**d**) Longitudinal section of wild-type root tip. (**e**) Longitudinal section of homozygous mutant root tip. (**f**) Transverse section of wild-type root tip. (**g**) Cross section of homozygous mutant root tip. Bars = 200 μm

A semi-dominant rice mutant, which was designated *nrtp1-D*, was isolated by screening for the short-root phenotype in an M_2_ ethyl methane sulfonate- (EMS-) treated population of rice cultivar Kasalath (*Oryza sativa ssp. indica*). The mutant was identified initially in the heterozygous state. Among self-pollinated offspring of the original mutant plant, 56/249 of the progeny exhibited a wild-type phenotype, and 63/249 of the progeny showed severe defects in plant growth, especially in terms of root development; these plants were seedling lethal. The remaining 130/249 of the progeny exhibited a mild mutant phenotype, with short roots, and the segregation rate among wild type, mild mutant and severe mutant phenotypes is 1:2:1 (*P* = 0.880). These mild mutants developed reproductive tissues and set seeds, with their offspring also segregating in a 1:2:1 ratio (Fig. 1a). These results indicate that the *nrtp1-D* phenotype segregates as a semi-dominant trait, homozygous mutant plants being seedling lethal.

The shoot and root lengths of both heterozygous and homozygous mutants were significantly shorter than those of the wild type, with a clear dosage effect (Fig. 1B). In particular, the homozygous *nrtp1-D* seedling was almost rootless, with very short roots less than 1 cm long. The short-root phenotype of both heterozygous and homozygous mutants was not due to a defect in root initiation, because the number of crown roots was similar between the wild type and the mutants (Fig. 1b). Although the mutant shoot was significantly shorter than that of the wild type, there were no other significant differences in both heterozygous and homozygous *nrtp1-D* leaves (Fig. 1c). To investigate the cellular pattern in root tips of the mutant, longitudinal and transverse sections of wild type and homozygous *nrtp1-D* mutants were compared. Although the meristem organization of the homozygous mutant seemed similar to that of the wild type, and no obvious defects in the quiescent center region were observed in the mutant, significant differences were observed in longitudinal sections of the root cap. Numerous layers of columella cells were observed in the root cap of the wild-type seedlings, and these cells contained starch granules, which appeared as small dots in the longitudinal sections (Fig. 1d). In the root cap of the homozygous mutant, however, the shapes of the columella cells were irregular, and they were not arranged in an orderly manner as they were in the wild type. In addition, we did not find starch granules in the columella cells of the homozygous mutant seedlings, indicating a loss of function in these cells (Fig. 1e). In the wild-type seedlings, the cells were arranged close together and they were stained easily by blue dye (Fig. 1f). In contrast, although the radial patterning appeared similar, the cells in the homozygous mutant seedlings were arranged loosely, with the cells in the outer layers (epidermis, exodermis and sclerenchyma layer) being wizened in appearance (Rebouillat et al. 2009). In addition, these cells did not stain as easily as did those of the wild type (Fig. 1g). These results indicated that the status of the cells in the root tip of the homozygous *nrtp1-D* mutant was abnormal.

### *NRTP1* encodes a CC-NB-LRR type protein

We isolated the mutated gene using a map-based cloning strategy. We first developed an F_1_ population by crossing the heterozygous mutant (*nrtp1-D*/*NRTP*) with wild-type ‘Nipponbare’ (*Oryza sativa ssp. japonica*). Segregation of the mild “short-root” phenotype in the F_1_ population displayed a ratio close to 1:1 (23 plants of the wild-type phenotype and 25 plants of the mild mutant phenotype, *P* = 0.885). The F_1_ seedlings with the mild mutant phenotype were self-pollinated to generate the F_2_ mapping population. Segregation in the F_2_ generation expressed a ratio close to 1:2:1. These results further support the fact that a single semi-dominant gene was responsible for the mutant phenotype.

**Fig. 2:**
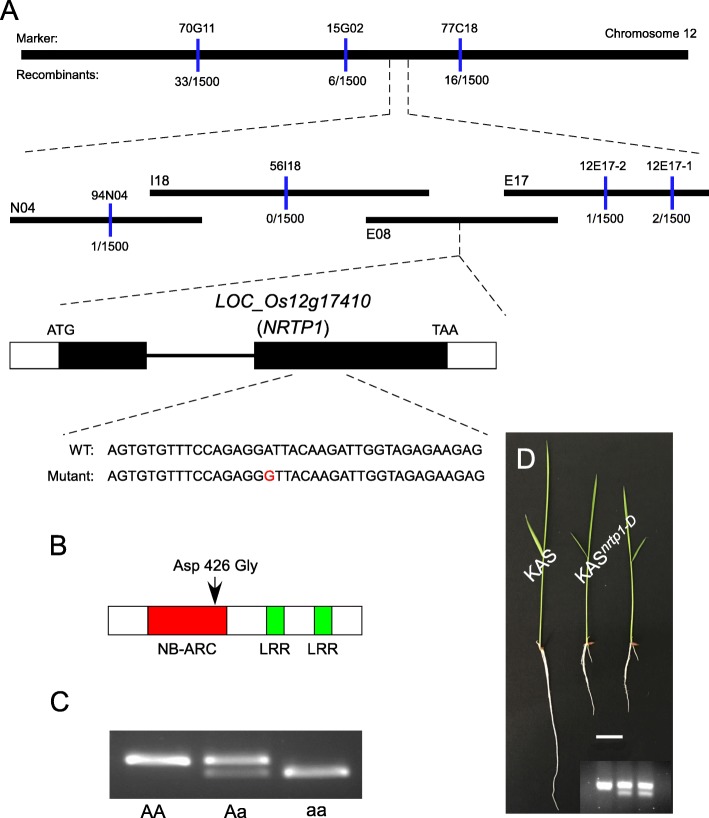
Map-based cloning of *NRTP1*. (**a**) Physical map of the *NRTP1* locus. The number of recombinants *per* 1500 meioses is indicated for each molecular marker represented by blue bars. N04, I18, E08 and E17 are abbreviations of BAC clones corresponding to OSJNBa0094N04, OSJNBa0056I18, OSJNBb0071E08 and OSJNBb0112E17, respectively. For the structure of the *NRTP1* gene, two boxes indicate the two exons and the line indicates the intron. (**b**) Structure of the NRTP1 protein. Arrow indicates the substitution in the 426-amino-acid protein in the *nrtp1-D* mutant. (**c**) A derived cleaved amplified polymorphic sequences (dCAPS) marker differentiates the wild type, heterozygous and homozygous mutant. (**d**) Complementation test. KAS^*nrtp1-D*^, two independent transgenic lines harboring mutated DNA fragmentexhibited the heterozygous *nrtp1-D* mutant phenotype. KAS, wild-type control. Insertion is the dCAPS marker confirmation of transgenic lines. Bar = 2 cm

Using PCR-based molecular markers, the location of the mutation was defined to a 170 kb region between markers 94 N04 and 12E17–2, and it co-segregated with marker 56I18 on chromosome 12. Although we enlarged the mapping population to 1500, we failed to narrow down the defined region. As a result, there were several candidate genes in this relatively wide region. This may due to the low recombination rate near the centromere within this region. We amplified and sequenced all the putative genes in this region from the homozygous mutant and found an A to G base pair substitution in the putative gene *LOC_Os12g17410*. Comparison of genomic and cDNA sequences of *LOC_Os12g17410* revealed that it consisted of two exons and one intron, and the mutation occurred in the second exon of *LOC_Os12g17410* (Fig. 2a). *LOC_Os12g17410* encoded a putative 916-amino-acid protein, containing a typical CC-NB-LRR structure. The mutation resulted in an Asp to Gly substitution in a non-conserved region of the NB-ARC domain (Figs. 2B, Additional files 3 and 4: Figures S1, S2). To facilitate future research, a dCAPS marker was developed to identify the mutation site (Additional file 5: Figure S3). Using this marker, all the homozygous mutants produced higher mobility bands than the wild type, while all of the heterozygous mutants produced double bands (Figs. 2c, Additional file 6: Figure S4).

To confirm our map-based cloning result, an 8 kb genomic fragment, containing the entire *LOC_Os12g17410* gene (including the native promoter and terminator) with the *nrtp1-D* point mutation, was cloned and transformed into wild-type ‘Kasalath’ plants via *Agrobacterium tumefaciens*-mediated transformation. Heterozygous *nrtp1-D* mutant-like morphology was observed in the transgenic plants (Fig. 2d). This result confirmed that *NRTP1* is *LOC_Os12g17410*, and that the mutation in *nrtp1-D* resulted in severe defects in plant development, especially with respect to root development.

### *NRTP1* is expressed preferentially in roots

**Fig. 3:**
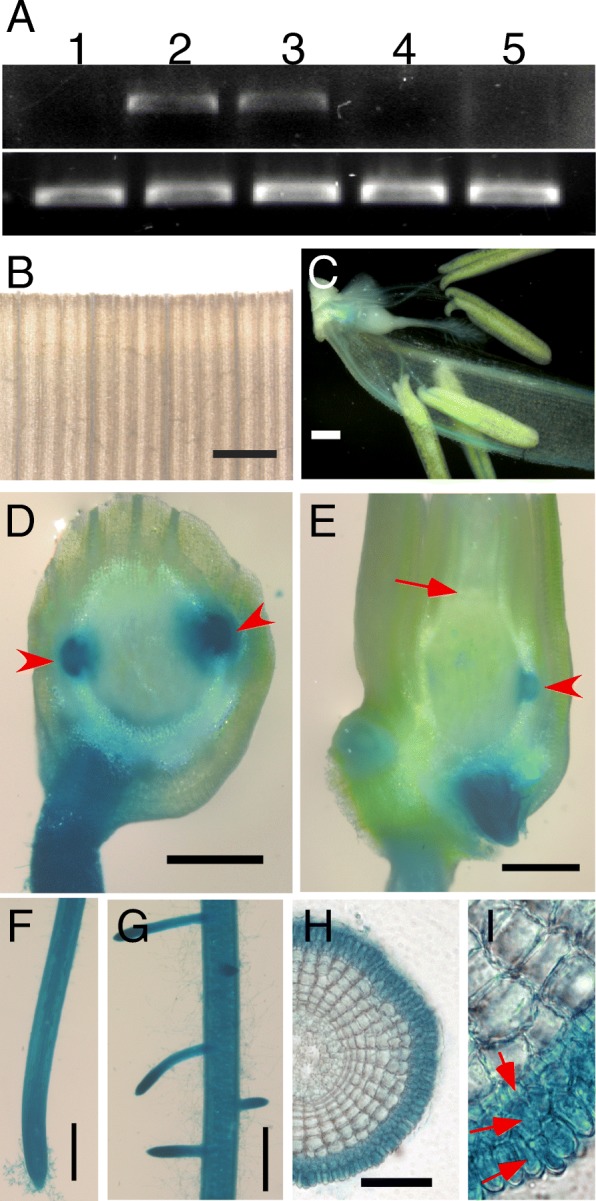
Tissue expression pattern of *NRTP1*. (**a**) RT-PCR analysis of *NRTP1* expression in different tissues. 1, shoot; 2, root; 3, stem base. 1, 2 and 3 are from 7-d-old seedlings. 4, stem; 5, young panicle. 4 and 5 are from mature plants. (**b**-**i**) GUS staining of *NRTP1p*-*GUS* transgenic plants. (**b**) Leaf; (**c**) Flower; (**d**) Transverse section of stem base. Arrow heads indicate crown root primordia; (**e**) Longitudinal section of stem base. Arrow indicates shoot apical meristem, arrow head indicates crown root primordia; (**f** and **g**) Root; (**h**) Transverse section of root; (**i**) Magnification of (**h**), arrows indicate three outermost cell layers stained by GUS. (**b**-**c**) mature plants, (**d**-**i**) 7-d-old seedlings. Bars = 500 μm in (**b**-**g**), bar = 100 μm in (**h**)

To study the expression pattern of *NRTP1* in different tissues of rice, we performed RT-PCR analysis in various tissues and found that the expression of *NRTP1* was only detected in roots and stem bases (Fig. 3a). To investigate the possible hormone induction of *NRTP1* expression in rice leaves, we examined the *NRPT1* expression after SA and JA treatments. RT-PCR analysis revealed that both SA and JA could slightly induce the expression of *NRTP1* in rice leaves (Additional file 7: Figure S5). To further analyze the expression of *NRTP1*, we generated *NRTP1p:GUS* transgenic lines. As expected, β-glucuronidase (GUS) staining was strongly detected in roots and stem bases (Figs. 3b to 3i). Longitudinal and transverse sections of stem bases revealed that only crown roots and crown root primordia were stained, while other tissues (including the shoot apical meristem) showed no GUS staining (Fig. 3d and e). Transverse section of roots revealed strong GUS staining in the three outermost layers of cells (Fig. 3h and i). These results indicated that *NRTP1* is strongly expressed in roots, and its expression is localized in the three outermost cell layers of rice roots.

### Ectopic expression of *NRTP1* does not affect the growth and development in rice

To explore the functions of NRTP1 in rice, we generated independent transgenic rice plants overexpressing wild-type *NRTP1* and mutated *nrtp1-D* under the control of the CaMV35S (35S) promoter, namely *35Sp:NRTP1* and *35Sp:nrtp1-D*. We generated eight lines of the transgenic *35Sp:NRTP1*. However, none of the lines showed any phenotypic differences from the wild type (Additional file 8: Figure S6). On the other hand, we failed to get transgenic rice overexpressing *nrtp1-D* (*35Sp:nrtp1-D*). This indicated the embryonic lethal condition, when *nrtp1-D* was not restricted to the root or root primordium.

### Expression of *nrtp1-D* in tobacco leaves induces necrotic lesions

**Fig. 4:**
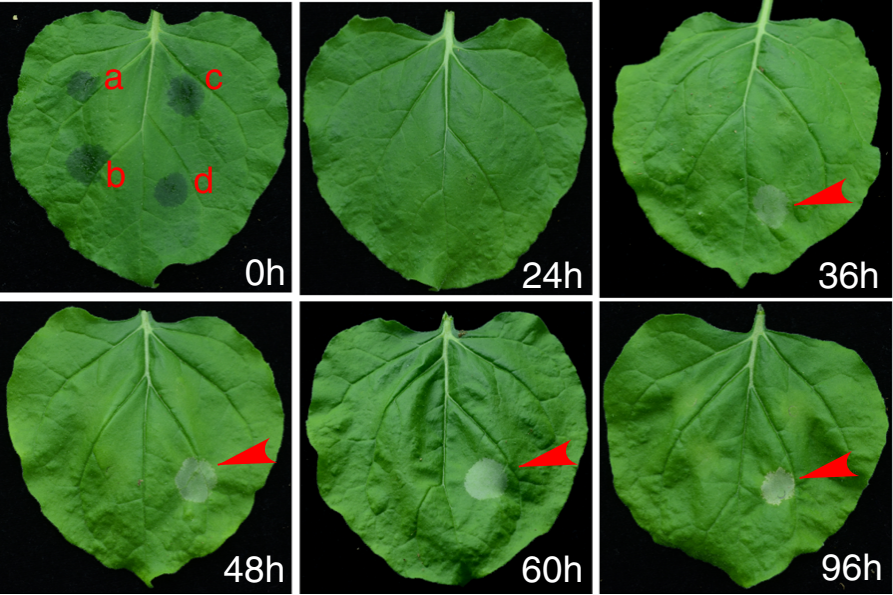
Expression of *nrtp1-D* in tobacco leaves induces necrotic lesions. Necrotic lesions were observed after transient expression of *nrtp1-D* in tobacco leaves. Each leaf was injected at four sites, with a, water; b, empty vector control; c, *NRTP1*; and d, *nrtp1-D*. Arrow heads indicate necrotic lesions caused by cell death

To examine the lethal effect of *nrtp1-D*, we transiently expressed *nrtp1-D* in tobacco leaves by *Agrobacterium*-mediated transient transformation. No significant differences were observed in the first 24 h after injection. However, necrotic lesions developed at a number of the sites expressing *nrtp1-D* (12/20) 36 h after injection. At 48 h after injection, all sites expressing *nrtp1-D* developed necrotic lesions (20/20). On the other hand, sites injected with wild-type *NRTP1* or with the empty vector control did not show any necrotic lesions, even 96 h after injection (Fig. 4).

### Transcriptome analysis of homozygous *nrtp1-D*

**Fig. 5:**
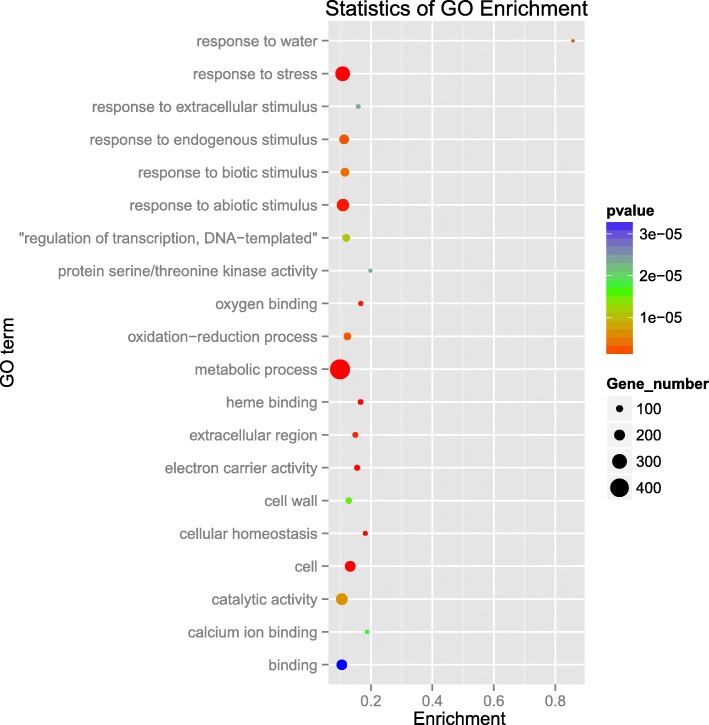
GO enrichment of differentially expressed genes

To further investigate the characteristics of the *nrtp1-D* mutant, the transcriptome of homozygous *nrtp1-D* rice was analyzed. The whole plant of 7-d-old homozygous *nrtp1-D* mutant and wild-type seedlings were harvested and three biological replicates were carried out. A total of 2819 genes exhibited altered expression level in the *nrtp1-D* mutant, compared to the wild type. The expressions of 1300 genes were up-regulated, while 1519 genes exhibited down-regulated expression in the *nrtp1-D* mutant (Additional files 9 and 10: Table S3, Table S4). A GO enrichment analysis showed that the largest proportion of differentially expressed genes (DEGs) was enriched with respect to the metabolic process. In addition, many DEGs were enriched with respect to the responses to different stresses (Fig. 5, Additional file 11: Table S5). A KEGG enrichment analysis showed that, except for the plant-pathogen interaction, most of the DEGs were enriched with respect to the pathways of metabolism, biosynthesis and degradation of different primary or secondary metabolic products (Additional files 12 and 13: Figure S7, Table S6). Further analysis revealed that many genes involved in the catabolic pathway were down-regulated. In contrast, many genes involved in the anabolic pathway were up-regulated. Nine percent of the genes in the plant-pathogen interaction pathway were up-regulated in the mutant, while 12% of the genes were down-regulated (Additional files 14 and 15: Figure S8, Table S7).

### Elevated NO levels in homozygous *nrtp1-D* mutant roots

**Fig. 6:**
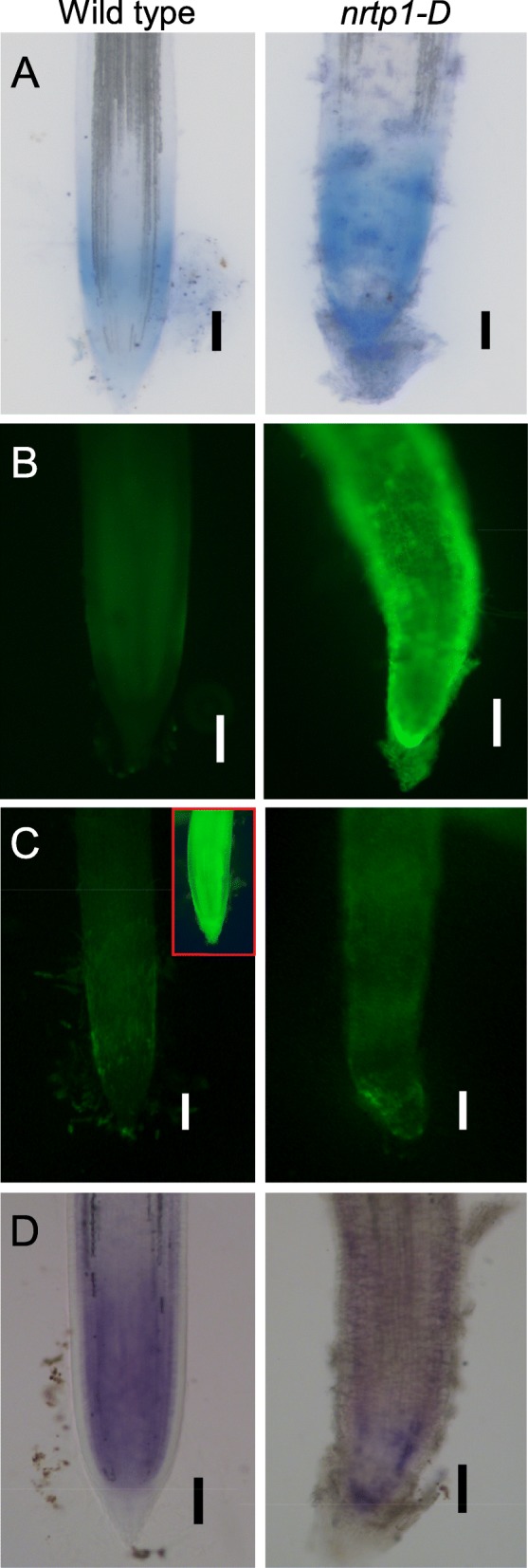
Staining for dead cells, NO and ROS in *nrtp1-D* mutant roots. 7-d-old seedling root tips were stained by (**a**) Evans blue (to stain dead cells), (**b**) 4-amino-5-methylamino-2′,7′-difluorofluorescein diacetate (to detect NO levels), (**c**) 3′-(ρ-hydroxyphenyl) fluorescein (to detect ROS levels) and (**d**) Nitro blue tetrazolium (to detect H_2_O_2_ and O_2_^.-^ levels), respectively. Insertion with red frame, positive control. Bars = 100 μm

In order to explore the status of homozygous *nrtp1-D* roots, 7-d-old seedling roots were stained with Evans blue. Large patches of stained cells were observed in *nrtp1-D* roots, indicating the occurrence of cell death in *nrtp1-D* root tips (Fig. 6A). Next, the NO-specific fluorescent probe 4-amino-5-methylamino-2′,7′-difluorofluorescein diacetate (DAF-FM DA) was used to investigate the endogenous NO levels in root tips. The fluorescence intensity was much higher in the *nrtp1-D* mutant than in the wild type, indicating an elevated NO level in *nrtp1-D* mutant roots (Fig. 6B). The ROS levels in root tips were also measured, using 3′-(ρ-hydroxyphenyl) fluorescein (HPF). The ROS levels in *nrtp1-D* root tips were not markedly higher than in the wild type (Fig. 6C). Specifically, H_2_O_2_ and O_2_^.-^ levels were measured by nitro blue tetrazolium (NBT) staining. H_2_O_2_ and O_2_^.-^ levels may even have been lower in homozygous *nrtp1-D* root tips than in wild-type root tips (Fig. 6D). We also measured the NO and H_2_O_2_ contents independently, and similar results were achieved (Additional file 16: Figure S9).

## Discussion

Many lesion-mimic mutants have been isolated in rice, and almost all of these lesions were observed on the leaf blades or sheaths (Shen et al. 2014; Takahashi et al. 1999; Tang et al. 2011; Wu et al. 2008; Yin et al. 2000). In this study, we describe a semi-dominant mutant, *nrtp1-D*. Heterozygous mutants exhibited a mild short-root phenotype, while homozygous mutants showed an extreme short-root phenotype. The homozygous *nrtp1-D* exhibited defense-related phenotypes in roots, including cell death and accumulation of NO in roots. Map-based cloning revealed that *NRTP1* encodes a typical CC-NB-LRR-type protein, of a type which is widely accepted to be involved in plant defense response (Lee and Yeom 2015).

Intramolecular interaction between NB-ARC domain and LRR domain within the CC-NB-LRR-type protein is essential for the protein to keep an auto-inhibited state (Takken and Goverse 2012). A number of previous experiments had shown that mutations in the NB-ARC domain may disrupt this interaction and resulted in auto-activation of defense response in plants (Bendahmane et al. 2002; Tang et al. 2011). Expression of the mutated *nrtp1-D* gene in wild-type ‘Kasalath’ resulted in altered morphology similar to that exhibited by the *nrtp1-D* heterozygote. Thus, the point mutation in *nrtp1-D* is responsible for the mutant phenotype, and it probably caused an auto-activation of the defense response in rice roots.

The expression of the mutated *nrtp1-D* gene caused cell death in situ in tobacco leaves. This showed that a common signaling cassette exists in evolutionarily divergent monocots and dicots. While plants have evolved hundreds of *R* genes (McHale et al. 2006), it is unlikely they have evolved a corresponding number of signaling pathways to couple with these R proteins. Thus, it is possible that plants utilize a conserved signaling cassette downstream of R-protein-mediated signaling. Consistent with this, a chaperone complex was reported to facilitate the correct folding of R proteins in pathogen recognition and signal transduction, and this chaperone is structurally and functionally conserved (Shirasu 2009). The failure to generate transgenic rice over-expressing the mutated *nrtp1-D* may be due to the cell death induced by the expression of *nrtp1-D* in situ, leading to embryonic lethality in the transgenic rice.

Transcriptome analysis revealed that, besides genes directly involved in the plant-pathogen interaction, gene expression levels in several other pathways were also altered in the homozygous *nrtp1-D* mutant. For instance, many genes involved in phenylpropanoid biosynthesis were upregulated in the mutant. Phenylpropanoids are widely involved in plant resistance at several levels, by providing building units of physical barriers, by synthesizing an array of antibiotic compounds and by producing signal molecules implicated in the mounting of plant resistance (La Camera et al. 2004). We also found up-regulation of genes involved in sugar metabolism in the mutant. Up-regulation of genes involved in sugar metabolism was observed in an early study of plant defense research, using microarray analysis (Schenk et al. 2000). Since then, a number of research studies have revealed the multifunctional roles of sugars in plant defense responses (Morkunas and Ratajczak 2014). In addition, many genes in the stilbenoid, diarylheptanoid and gingerol biosynthesis pathway were down-regulated in the *nrtp1-D* mutant, and similar results were also reported in the defense responses of other plant species (Xu et al. 2015; Zhang et al. 2014). These results supported the hypothesis that auto-activation of plant defense responses occurred in the *nrtp1-D* mutant. On the other hand, we also found down-regulation of genes that were involved in many catabolic pathways. Some of the pathways, such as pathways for the degradation of polycyclic aromatic hydrocarbons and bisphenols, have not previously been reported to be associated with plant defense responses. This may be due to the specificity of the defense response in rice roots. Alternatively, these unusual gene expression changes may be due to the cascade effect emanating from severe defects in root functions, as a result of cell death in mutant roots.

NO and ROS signaling pathways are closely connected with plant biotic interactions, including symbiotic interactions, herbivore attacks and disease responses (Scheler et al. 2013). Moreover, NO and ROS synthesis are considered to be a routine requirement for plant cells to undergo PCD during plant defense responses (Wang et al. 2013). After the recognition of pathogen attack, NO accumulation occurred concomitantly with the production of ROS at the site of invasion (Romero-Puertas et al. 2004). However, in our experiments, we found that elevated NO levels were accompanied by almost unchanged ROS levels in homozygous *nrtp1-D* mutant root tips (Fig. 6). To explain this unusual phenomenon, we propose that the structure of the root, compared with the leaf, is complex and fragile (Ni et al. 2014b; Ni et al. 2014c). For instance, cell death surrounding infection sites in a leaf would not destroy the entire leaf, because water and nutrients could still be transported by alternative routes near the infection sites by the complex leaf vein network. However, the situation is different for roots. If cell death occurred surrounding the infection sites, it may spread into the vascular cylinder nearby, and this would endanger the entire root structures below the infection site, resulting in total interruption of water and nutrient transport at the infection sites. Thus, we speculate that a different (compared to that in leaves) and precisely controlled strategy of cell death may be used in the root defense responses. Interestingly, it has been shown that, in addition to the reinforcing relationship between NO and ROS, NO can also scavenge ROS to protect plant cells from further damage under certain conditions (Beligni et al. 2002; Crawford and Guo 2005). Furthermore, high concentrations of NO would abolish ROS synthesis by S-nitrosylation of NADPH oxidase to regulate a negative feedback loop, limiting the HR responses (Yun et al. 2011). Thus, our results may indicate a different mechanism of defense response which operates in plant roots, compared to leaves.

## Conclusions

We isolated and characterized a semi-dominant short-root rice mutant *nrtp1-D*. Map-based cloning revealed that *NRTP1* encoded a typical CC-NB-LRR type protein and the mutation caused an amino acid substitution in the NB-ARC domain, which might have caused constitutive auto-activation of the NRTP1 protein. As expected, transient expression of the mutated *nrtp1-D* in tobacco leaves induced the formation of necrotic lesions. Transcriptome analysis revealed that many typical defense response genes were expressed in *nrtp1-D*, although we also found differential expression of genes not previously reported to be associated with plant defense. The level of NO, but not ROS, was increased in *nrtp1-D* roots. This is different from a previous report, where auto-activation of the NLS1 R protein caused constitutive activation of defense responses, accompanied by increased ROS levels in rice leaves (Tang et al. 2011). Thus, our results indicate that, in addition to the mechanism of defense response common to both roots and shoots, a novel pathway may also exist in rice roots, which is different from that in shoots.

## Additional files

Additional file 1: **Table S1.** Primers used in this research. (XLSX 11 kb)

Additional file 2: **Table S2.** Summary of sequencing data for each sample. Valid data were obtained after removing adaptor sequences and low-quality reads (Reads with more than 5% unidentified bases or more than 20% low-quality bases). Q20 (%), ratio of reads with quality more than Q20. Q30 (%), ratio of reads with quality more than Q30. (XLSX 10 kb)

Additional file 3: **Figure S1.** Protein sequence of NRTP1. Domains are marked by different colors. A substitution from Asp (D) to Gly (G) is indicated by an arrow. (PDF 62 kb)

Additional file 4: **Figure S2.** Amino acid alignment of NRTP1 with other CC-NB-LRR proteins identified in rice. Substitution from D to G in NRTP1 is marked by a red arrow. Substitutions in NSL1 are marked in red. (PDF 350 kb)

Additional file 5: **Figure S3.** dCAPS marker for molecular identification. (A) DNA sequences of wild type and mutant. The mutated base is marked in red. The dis-matched base to the primer 17410-L is marked by red fork. (B) PCR products of wild type and mutant sequences. A point mutation is introduced after the PCR amplification (marked in green), resulting an *Eco*91I recognition site in mutant. (PDF 62 kb)

Additional file 6: **Figure S4.** Co-segregation of phenotype and genotype. All the homozygous mutants produced higher mobility bands than the wild type. All the heterozygous mutants produced double bands. Bar = 2 cm. (PDF 2864 kb)

Additional file 7: **Figure S5.** RT-PCR analysis of *NRTP1* expression after SA and JA treatments. Untreated roots are positive control. Numbers on the right are cycles in PCR. (PDF 166 kb)

Additional file 8: **Figure S6.** Phenotypes of transgenic plants overexpressing *NRTP1*. (A) Comparison of phenotypes between wild type (WT) and transgenic plants overexpressing *NRTP1*. Line 1 and line 2 indicate independent transgenic lines. RT-PCR confirmation is shown at the bottom of the figure; the first row is the expression of *NRTP1*, the second row is the expression of the *OsActin* control. (PDF 1354 kb)

Additional file 9: **Table S3.** Expression levels of all genes. The expression levels (FPKM) of each gene in three replicates are shown. (XLSX 5889 kb)

Additional file 10: **Table S4.** Differentially expressed genes in *nrtp1-D*. The expression levels of each gene in the table are the average of three replicates. (XLSX 526 kb)

Additional file 11: **Table S5.** GO classification of differentially expressed genes. (XLSX 892 kb)

Additional file 12: **Figure S7.** KEGG enrichment of differentially expressed genes. (PDF 40 kb)

Additional file 13: **Figure S6.** KEGG classification of differentially expressed genes. (XLS 247 kb)

Additional file 14: **Figure S8.** Down-regulated and up-regulated genes in the most significantly enriched pathways in *nrtp1-D*. (PDF 79 kb)

Additional file 15: **Table S7.** Down-regulated and up-regulated genes in different pathways. (XLSX 65 kb)

Additional file 16: **Figure S9.** NO and H_2_O_2_ contents in leaves and root tips of 7-d-old wild type and homozygous *nrtp1-D*. Asterisks indicate significant differences (*P* < 0.01; Student’s *t*-test). FW, fresh weight. Data of independent experiments are shown (mean ± SD; *n* = 3). (PDF 183 kb)

## Abbreviations

35S: CaMV35S
CC: coiled-coil
CNL: CC-NB-LRR
DAF-FM DA: 4-amino-5-methylamino-2′,7′-difluorofluorescein diacetate
dCAPS: Derived cleaved amplified polymorphic sequences
EMS: Ethyl methane sulfonate
ETI: Effector-triggered immunity
GO: Gene Ontology
GUS: β-glucuronidase
HPF: 3′-(ρ-hydroxyphenyl) fluorescein
HR: Hypersensitive response
KEGG: Kyoto Encyclopedia of Genes and Genomes
NADPH: Nicotinamide adenine dinucleotide phosphate
NB-ARC: Nucleotide-Binding adaptor shared by Apaf1, certain R genes and CED4
NB-LRR: Nucleotide-binding site leucine-rich repeat
NBT: Nitro blue tetrazolium
NO: Nitric oxide
NPR1: Non-expresser of pathogen related gene 1
NRTP1: Necrotic root tip 1
PAMP: Pathogen-associated molecular pattern
PCD: Programed cell death
PTI: PAMP-triggered immunity
R: Resistance
ROS: Reactive oxygen species
TIR: toll/interleukin-1 receptor
TNL: TIR-NB-LRR
