# Supplementary material for: A semi-dominant mutation in a CC-NB-LRR-type protein leads to a short-root phenotype in rice
Source: Rice (N Y). 2018 Oct 3;11:54. doi: 10.1186/s12284-018-0250-1 (PMC6170248; doi:10.1186/s12284-018-0250-1)
Supplement: Supplementary file 3 — Figure S1. Protein sequence of NRTP1. Domains are marked by different colors. A substitution from Asp (D) to Gly (G) is indicated by an arrow. (PDF 62 kb) [file 12284_2018_250_MOESM3_ESM.pdf]

Figure S1

MAGAIVSVSTGALSTLLPKLSLLIQGEYKLLKGVKGGISFLRDELTSMHTLLVKLANNEEKLDEQV

KDWRNKVRELSYDIEDCIDLFLHKVSSSNAKASLVRKTAAKIRKLWSRHKIANLIEELKARVIEES

DRRSRYNFDEVADKFSHVQIDPRLPALYVEAEKLVGIDGPREKIIRWLEKDESLKLKIVCIVGFGG

LGKTTLANQVYHKIKGQFDCFSFVPVSRNPNILKILADMLKELGSNVDTSDDQRQLISKLRTFLE

HQRYLVIIDDIWSTQAWEVIKCVLPENNLRSRIISTTRNSDVATSCCSSLAGYIHNIQPLNDQDS

QKLFFKRIFGDESACPPYLEQVSHGIISKCHGLPLALISIASLLAGKSRMKEQWEQVYNSIGFAFS

HQGIRDILLLSYYDLPIHLKTCLLYLSVFPE**D**YKIGREELIWRWIAEGFISEVKGQTLDQVAENYLN

↓

G

NB-ARC domain

DLVNRSMIQPVDIKYDGRADACKLHDMVLDLIISLSTQENFTTIVEGQQYKCSSNKIRRISIHSKC

LEDEVMQEIMTNCLQVRSISFYGLQDQETSLLPTLNSLRVLAFENWHHRGSKSIKHLGRFFQLT

YLRINSRGIYELPEQIGGLQNLLTLDIRGSEVKKLPSTIGCLKNLVRLLVNDYVELPNEIGDLQALQ

LRR domain

QLSYADNYNSIVFVEQLKRLANLREIGIQLHGSAQLGDHDMARYMEALKSSLAVMGKQGLQS

LEISYGHDMVIGEKLMDLLCYSPCLRKLVIDSSRISRLSKQMALLVNLRHLDIGVSNIKQGDLCVL

LRR domain

GSIPTLLFVRLFVENGPDERLAIISHQFRCLKQFIFISLGGGLEMLFLQEAMPELRRLSLSFSAEET

DCKMGFEFSFKHLASLEHLKVTIDCGDATRSRVEAAEASVRNAASAHPGCPRIEMINRYSKTS

Figure S1. Protein sequence of NRTP1. Domains are marked by different colors. A substitution from Asp (D) to Gly (G) is indicated by an arrow.
