# Supplementary material for: A semi-dominant mutation in a CC-NB-LRR-type protein leads to a short-root phenotype in rice
Source: Rice (N Y). 2018 Oct 3;11:54. doi: 10.1186/s12284-018-0250-1 (PMC6170248; doi:10.1186/s12284-018-0250-1)
Supplement: Supplementary file 4 — Figure S2. Amino acid alignment of NRTP1 with other CC-NB-LRR proteins identified in rice. Substitution from D to G in NRTP1 is marked by a red arrow. Substitutions in NSL1 are marked in red. (PDF 350 kb) [file 12284_2018_250_MOESM4_ESM.pdf]

Figure S2

```
                          *        20         *        40         *        60         *        80         *       100         *
NRTP1    : --MAGAIVSVSTGALSTLLPKLSLLIQG---------EYKLLKGVKGGISFLRDELTSMHTLLVKLANNE----EKLDEQVKDWRNKVRELSYDIEDCIDLFLHKVSS----
NSL1     : --MEAAIVSASTGVMRSLLAKLTNLLGG---------EYKLLKWLRREMEFLESELRSMSIFLERLEDT-----QKLHPQMKDWRDRVRELAYDIEDCIDDFILQLDS----
OsPi-km2 : ---MELVVGASEATMKSLLGKLGNLLAQ---------EYALISGIRGDIQYINDELASMQAFLRDLSNVPEG--HSHGHRMKDWMKQIRDIAYDVEDCIDDFAHRLPQ----
OsPi-b   : --MEATALSVGKSVLNGALGYAKSAFAE---------EVALQLGIQKDHTFVADELEMMRSFMMEAHEE-----QDNSKVVKTWVKQVRDTAYDVEDSLQDFAVHLKRPSWW
OsPi-ta  : --MAPAVIASQGVIMRSLTSKLDSLILQPPEPPPPAQPSSIRKGERKKILLLRGDLRHLLDDYYLLVEPPSDTAPPPDSTAACWAKEVRELSYDVDDFLDELTTQLLH----
OsPi36   : -------MEFVTGAMSSLIPKLGELLKE---------KYNLQKNIRGKIESLSAELRRAQAVIRMVSEVPQ---DQQNELVKLWASDLREASYDMEDIIDAFLVHVDD----
OsPi-d3  : --MAEGVVGSLIVKLGDALASEAVEVAKSLLG----LEGSALKRLFSEIREVKGELESIHAFLQAAERFK-----DADETTSAFVKQVRSLALSIEDVVDEFTYELGE----
OsPi-km1 : MEAAAMAVTAATGALAPVLVKLAALLDDG--------ECNLLEGSRSDAEFIRSELEAVHSLLTPNILGRMG--DDDAACKDGLIAEVRELSYDLDDAVDDFLELNFEQRRS
OsBph14  : --MAELMATMVVGPLLSMVKDKASSYLLE--------QYKVMEGMEEQHEILKRKLPAILDVIADAEEQAAK----HREGVKAWLEALRKVAYQANDVFDEFKYEALRR---
OsXa1    : --MEEVEAGWLEGGIRWLAETILDNLDADKLD-----EWIRQIRLAADTEKLRAEIEKVDGVVAAVKGRAIG-----NRSLARSLGRLRGLLYDADDAVDELDYFRLQQQVE
                         6                                    6   e6                                 6R    yd  D  d f

                     160         *       180         *       200         *       220         *       240         *       260
NRTP1    : SLVRKTAAKIRKLWSRHKIANLIEELKARVIEESDRRSRYNFDEVADKFSH-------------------------------------------------------------
NSL1     : GFG-------QKLLASRRIGHMIRELKARVMEESERQRRYMLDGLASGPSVR------------------------------------------------------------
OsPi-km2 : SFLLTKIYELWTWWPRRVIASNIAQLKVRAQQIADRRSRYGVNNPEHLDSSSSA----------------------------------------------------------
OsPi-b   : LIKGSAKATINSTEQSSVIATAIFGIDDARRAAKQDNQRVDLVQLINSEDQDLKVIAVWGTSGDMGQTTIIRMAYENPDVQIRFPCRAWVRVMHPFSPRDFVQSLVNQLHAT
OsPi-ta  : MISSMIARLRGELNRRRWIADEVTLFRARVKEAIRRHESYHLGRRTSSSRPREEDDDDD-----------------------------------------------------
OsPi36   : RLGKQVKKLFKKTKHRVTIADAIQEMEKKLLEIDARHGRYPVDNIVIPARP-------------------------------------------------------------
OsPi-d3  : MGMAVALKRMCKMGTWSRLAGNLQDIKVNLKNAAERRIRYDLKGVERGAKSTAG----------------------------------------------------------
OsPi-km1 : GLPPPDAELVGMDKRMEELTKLLEQGSNDASRWRKRKPHFPLRKTGLKQKIVIKVAMEG-----------------------------------------------------
OsBph14  : KLIPTHNRILFSYRMGNKLRMILNAIEVLIEEMNAFRFKFRPEPPMSSMKWR------------------------------------------------------------
OsXa1    : DVPEAVAAGSSKKRSKAWEHFTTVEFTADGKDSKARCKYCHKDLCCTSKNGTSALRNHLN----------------------------------------------------
                                                  r

                          *       320         *       340         *       360         *       380         *       400         *
NRTP1    : ---------------------------------------------VQIDPRLPALYVEAEKLVG-------------------------------IDGPREKII-RWLEK--
NSL1     : ---------------------------------------------VKVDPRLSALYVDEDRLVG-------------------------------IDAPRDEIIGRLLDKRR
OsPi-km2 : --------------------------------------------RTRAVNYEIAEYQVTSPQIIG-------------------------------IKEP-VGMKTVMEELEV
OsPi-b   : IEEWDQIKKCFQKCRKGSRIIVSSTQVEVASLCAGQESQASELKQLSADQTLYAFYDKGSQIIEDSVKPVSISDVAITSTNNHTVAHGEIIDDQSMDADEKKVARKSLTRIR
OsPi-ta  : --------------------------------------------REDSAGNERRRFLSLTFGMDD-------------------------------AAVHGQLVGRDISMQKL
OsPi36   : ---------------------------------------------TPIDPHILNIEKMANNLVG-------------------------------IDEPRDELIKMLFQHER
OsPi-d3  : --------------------------------------------RRSSNWRSDSVLFKREDELVG-------------------------------IEKKRDLLMKWVKDE--
OsPi-km1 : -----------------------------------------NNCRSKAMALVASTGGVDSVALVGDLRDKIEVVG----------------------YGIDPIKLISALRKKVG
OsBph14  : ---------------------------------------------KTDSKISDLSLDIANNSR--------------------------------KEDKQEIVSRLLVP--
OsXa1    : ------------------------VCKRKRVTSTDQPVNPSSAGEGASNATGNSVGRKRMRMDGTSTHHEAVSTHPWN---KAELSNRIQCMTHQLEEAVNEVMRLCRSSSS
                                                                                                                   6
```

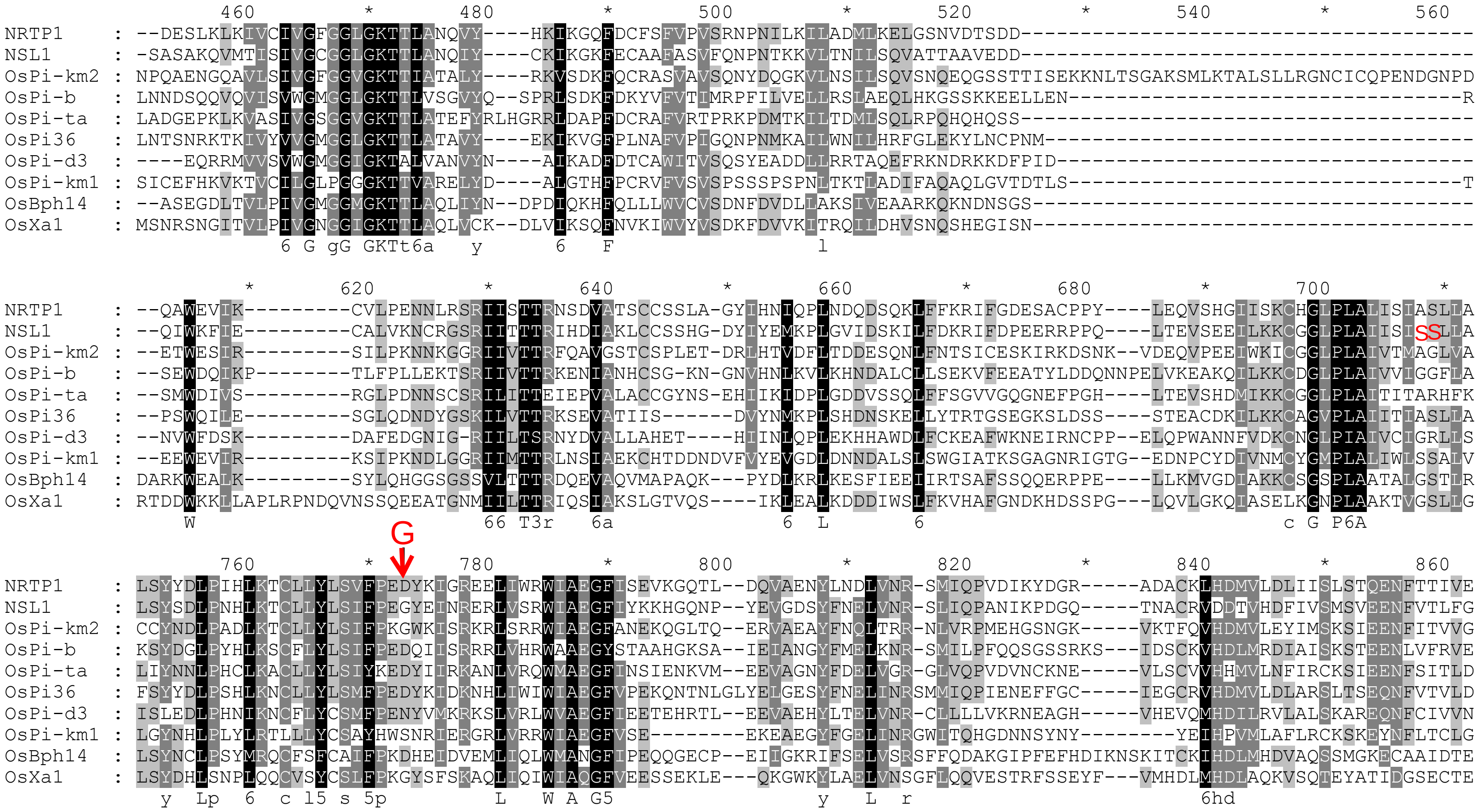
460 * 480 * 500 * 520 * 540 * 560
NRTP1 : --DESLKLKIVCIVGFGGLGKTTLANQVY----HKIKGQFDCFSFVPVSRNPNILKILADMLKELGSNVDTSDD--------------------------------------
NSL1 : -SASAKQVMTISIVGCGGLGKTTLANQIY----CKIKGKFECAAFASVFQNPNTKKVLTNILSQVATTAAVEDD--------------------------------------
OsPi-km2 : NPQAENGQAVLSIVGFGGVGKTTIATALY----RKVSDKFQCRASVAVSQNYDQGKVLNSILSQVSNQEQGSSTTISEKKNLTSGAKSMLKTALSLLRGNCICQPENDGNPD
OsPi-b : LNNDSQQVQVISVWGMGGLGKTTLVSGVYQ--SPRLSDKFDKYVFVTIMRPFILVELLRSLAEQLHKGSSKKEELLEN---------------------------------R
OsPi-ta : LADGEPKLKVASIVGSGGVGKTTLATEFYRLHGRRLDAPFDCRAFVRTPRKPDMTKILTDMISQLRPQHQHQSS--------------------------------------
OsPi36 : LNTSNRKTKIVYVVGMGGLGKTTLATAVY----EKIKVGFPLNAFVPIGQNPNMKAILWNILHRFGLEKYLNCPNM------------------------------------
OsPi-d3 : ----EQRRMVVSVWGMGGIGKTALVANVYN----AIKADFDTCAWITVSQSYEADDLLRRTAQEFRKNDRKKDFPID-----------------------------------
OsPi-km1 : SICEFHKVKTVCILGLPGGGKTTVARELYD----ALGTHFPCRVFVSVSPSSSPSPNLTKTLADIFAQAQLGVTDTLS---------------------------------T
OsBph14 : --ASEGDLTVLPIVGMGGMGKTTLAQLIYN--DPDIQKHFQLLLWVCVSDNFDVDLLAKSIVEAARKQKNDNSGS-------------------------------------
OsXa1 : MSNRSNGITVLPIVGNGGIGKTTLAQLVCK--DLVIKSQFNVKIWVYVSDKFDVVKITRQILDHVSNQSHEGISN-------------------------------------
6 G gG GKTt6a y 6 F l
* 620 * 640 * 660 * 680 * 700 *
NRTP1 : --QAWEVIK---------CVLPENNLRSRIISTTRNSDVATSCCSSLA-GYIHNIQPLNDQDSQKLFFKRIFGDESACPPY----LEQVSHGIISKCHGLPLALISIASLLA
NSL1 : --QIWKFIE---------CALVKNCRGSRIITTTRIHDIAKLCCSSHG-DYIYEMKPLGVIDSKILFDKRIFDPEERRPPQ----LTEVSEEILKKCGGLPLAIISISSLLA
OsPi-km2 : --ETWESIR---------SILPKNNKGGRIIVTTRFQAVGSTCSPLET-DRLHTVDFLTDDESQNLFNTSICESKIRKDSNK--VDEQVPEEIWKICGGLPLAIVTMAGLVA
OsPi-b : --SEWDQIKP--------TLFPLLEKTSRIIVTTRKENIANHCSG-KN-GNVHNLKVLKHNDALCLLSEKVFEEATYLDDQNNPELVKEAKQILKKCDGLPLAIVVIGGFLA
OsPi-ta : --SMWDIVS---------RGLPDNNSCSRILITTEIEPVALACCGYNS-EHIIKIDPLGDDVSSQLFFSGVVGQGNEFPGH----LTEVSHDMIKKCGGLPLAITITARHFK
OsPi36 : --PSWQILE---------SGLQDNDYGSKILVTTRKSEVATIIS------DVYNMKPLSHDNSKELLYTRTGSEGKSLDSS----STEACDKILKKCAGVPLAIITIASLLA
OsPi-d3 : --NVWFDSK---------DAFEDGNIG-RIILTSRNYDVALLAHET----HIINLQPLEKHHAWDLFCKEAFWKNEIRNCPP--ELQPWANNFVDKCNGLPIAIVCIGRLLS
OsPi-km1 : --EEWEVIR---------KSIPKNDLGGRIIMTTRLNSIAEKCHTDDNDVFVYEVGDLDNNDALSLSWGIATKSGAGNRIGTG--EDNPCYDIVNMCYGMPLALIWLSSALV
OsBph14 : DARKWEALK---------SYLQHGGSGSSVLTTTRDQEVAQVMAPAQK---PYDLKRLKESFIEEIIRTSAFSSQQERPPE----LLKMVGDIAKKCSGSPLAATALGSTLR
OsXa1 : RTDDWKKLLAPLRPNDQVNSSQEEATGNMIILTTRIQSIAKSLGTVQS----IKLEALKDDDIWSLFKVHAFGNDKHDSSPG---LQVLGKQIASELKGNPLAAKTVGSLLG
W 66 T3r 6a 6 L 6 c G P6A
G
760 * 780 * 800 * 820 * 840 * 860
NRTP1 : LSYYDLPIHLKTCLLYLSVFPEDYKIGREELIWRWIAEGFISEVKGQTL--DQVAENYLNDLVNR-SMIQPVDIKYDGR-----ADACKLHDMVLDLIISLSTQENFTTIVE
NSL1 : LSYSDLPNHLKTCLLYLSIFPEGYEINRERLVSRWIAEGFIYKKHGQNP--YEVGDSYFNELVNR-SLIQPANIKPDGQ-----TNACRVDDTVHDFIVSMSVEENFVTLFG
OsPi-km2 : CCYNDLPADLKTCLLYLSIFPKGWKISRKRLSRRWIAEGFANEKQGLTQ--ERVAEAYFNQLTRR-NLVRPMEHGSNGK-----VKTFQVHDMVLEYIMSKSIEENFITVVG
OsPi-b : KSYDGLPYHLKSCFLYLSIFPEDQIISRRRLVHRWAAEGYSTAAHGKSA--IEIANGYFMELKNR-SMILPFQQSGSSRKS---IDSCKVHDLMRDIAISKSTEENLVFRVE
OsPi-ta : LIYNNLPHCLKACLLYLSIYKEDYIIRKANLVRQWMAEGFINSIENKVM--EEVAGNYFDELVGR-GLVQPVDVNCKNE-----VLSCVVHHMVLNFIRCKSIEENFSITLD
OsPi36 : FSYYDLPSHLKNCLLYLSMFPEDYKIDKNHLIWIWIAEGFVPEKQNTNLGLYELGESYFNELINRSMMIQPIENEFFGC-----IEGCRVHDMVLDLARSLTSEQNFVTVLD
OsPi-d3 : ISLEDLPHNIKNCFLYCSMFPENYVMKRKSLVRLWVAEGFIEETEHRTL--EEVAEHYLTELVNR-CLLLLVKRNEAGH-----VHEVQMHDILRVLALSKAREQNFCIVVN
OsPi-km1 : LGYNHLPLYLRTLLLYCSAYHWSNRIERGRLVRRWIAEGFVSE--------EKEAEGYFGELINRGWITQHGDNNSYNY--------YEIHPVMLAFLRCKSKEYNFLTCLG
OsBph14 : LSYNCLPSYMRQCFSFCAIFPKDHEIDVEMLIQLWMANGFIPEQQGECP--EIIGKRIFSELVSRSFFQDAKGIPFEFHDIKNSKITCKIHDLMHDVAQSSMGKECAAIDTE
OsXa1 : LSYDHLSNPLQQCVSYCSLFPKGYSFSKAQLIQIWIAQGFVEESSEKLE---QKGWKYLAELVNSGFLQQVESTRFSSEYF---VMHDLMHDLAQKVSQTEYATIDGSECTE
y Lp 6 c l5 s 5p L W A G5 y L r 6hd

```
                         *       920         *       940         *       960         *       980         *      1000         *
NRTP1         : TNCL---QVRSISFYGLQDQETSLLPT-----LNSLRVLAFENWHHRGSKSIK----HLGRFFQLTYLRINSR-GIYELPEQIGGLQNLLTLDIRGSE--VKKLPSTIGCLK
NSL1          : LVTS---QVRSLTLFAVEMP--SLLG------FGMLRVLDLEDCYALEDHHLT----NLERLVQLRYLSIRTS-PISELPKQIGQLQYLETLDLRATG--VEELPSTIGRLK
OsPi-km2      : LNLA---QVRSLTVFGNLNHVPFHSFN-----YGIIQVLDLEDWKGLKERHMT----EICQMLLLKYLSIRRT-EISKIPSKIQKLEYLETLDIRETY--VRDLPKSIVQLK
OsPi-b        : VDLS---RIRSLSLFG--DWKPFFVYGK----MRFIRVLDFEGTRGLEYHHLD----QIWKLNHLKFLSLRGCYRIDLLPDLLGNLRQLQMLDIRGTY--VKALPKTIIKLQ
OsPi-ta       : LRLS---QVRSMAFFGQVKCMPSIADYR----LLRVLILCFWADQEKTSYDLT----SISELLQLRYLKITGN-ITVKLPEKIQGLQHLQTLEADARA---TAVLLDIVHTQ
OsPi36        : MDMK---KVRSFVATECNNGNNSVAPPR----FQVLRVLSLDKCNGMEDYYIESILQYAGRLGHLRCLQLSSHTEFHRLPKELGDLKFLKILDLGDCGGTIEELPEELGLLT
OsPi-d3       : DHAP---HLRSLLLFQSSPNVSSLQSLP----KSMKLLSVLDLTDSSVDRLPK----EVFGLFNLRFLGLRRT-KISKLPSSIGRLKILLVLDAWKCK--IVKLPLAITKLQ
OsPi-km1      : MDVS---HTCSLVVLGDVARPKGIPFYM----FKRLRVLDLEDNKDIQDSHLQG--ICEQLSLRVRYLGLKGT-RIRKLPQEMRKLKHLEILYVGSTR--ISELPQEIGELK
OsBph14       : LICSRFKYLQNVSKYRSLRVLTTMWEGS----FLIPKYHHHLRYLDLSESEIKALPEDISILYHLQTLNLSRCLSLRRLPKGMKYMTALRHLYTHGCWS-LGSMPPDLGHLT
OsXa1         : MKVKSRSKLRSLVLIGQYDSHFFKYFKDAFKEAQHLRLLQITATYADSDSFLS----SLVNSTHLRYLKIVTEESGRTLPRSLRKYYHLQVLDIGYRFG-IPRISNDINNLL
                         rs                           l                         6  L 6        6P  6     L  L           6p  6  l

                      1060         *      1080         *      1100         *      1120         *      1140         *      1160
NRTP1         : YNSIVFVEQLKRLA-----------NLREIGIQLHGSAQLGDH-------------------------DMARYMEALKSSLAVMGKQGLQSLEISYGHDMVIGEKLMD----
NSL1          : MYSPDFLKELGQLT-----------NMRVLRVICDCDSFKG---------------------------DAGSCLENLASSLCNLGTYNLHSLFVDINGYGEDNFSLDT----
OsPi-km2      : PAKKGFLSQEKGKG-----------AMKALRVLSGIEIVEESSEVAAGLHQLTGLR--KLAIYKLNITKGGDTFKQLQSSIEYLGSCGLQTLAINDENSEFINSLGDMP---
OsPi-b        : LLCEMYGPLHKALARRDA--WTFACCVKFPSIMTGVHEEEGAMVPSG----IRKLK--DLHTLRNINVGRGNAILRDIGMLTGLHKLGVAGINKKNGRAFRLAISNLNKLES
OsPi-ta       : YIFTSIPKWTGKLN-----------NLRILNIAVMQISQDD------------------------------------LDTLKGLGSLTALSLLVRTAPAQRIVAANEG----
OsPi36        : VAVRHFVQELGNLR-----------ELRVLYAKIYIGLKDE---------------------------SMQRDFLQSLGCLHKVHTMNIYSIEVRECTRPDAAGSVSCPRL-
OsPi-d3       : LLLMEASSQMVHHLG----------SLVELRTFRISKVR-------------------------------SCHCEQLFMAITNMIHLTRLGIQADSSQEVLHLESLKP----
OsPi-km1      : PPQVGKLQNLKIMCVRSTGVRELPKEIGELNHLQTLDVRNTRVR------------------------ELPWQAGQISQSLRVLAGDSGDGVRLPEGVCEALINGIPGATR-
OsBph14       : VTKADAKAANLGKKEKLTKLTLIWTDQEYKEAQSNNHKEVLEGLTPHEG--------LKVLSIYHCGSSTCPTWMNKLRDMVGLELNGCKNLEKLPPLWQLPALQVLCLEG-
OsXa1         : NLSGFEVTQLKSMNKLVQLSVSQLENVRTQEEACGAKLKDKQHLEKLHLSWKDAWNGYDSDESYEDEYGSDMNIETEGEELSVGDANGAQSLQHHSNISSELASSEVLEGLE
                                                                                6          6

                         *      1220         *      1240         *      1260         *      1280         *      1300         *
NRTP1         : KQMALLVNLRHLDIGVSNIKQGD--LCVLGSIPTLLFVRLFVENG---------PDERLAIISHQFRCLKQFIFISL------GGGLEMLFLQEAMPELRRLSLSFSAE---
NSL1          : NWVGSLINLEELVLYVNKIWQED--FELLGHMPALSSLTIYSNTA---------LQGRIIISG--FHSTKFFKFYCN------PAG--LTFDAGSLLKLECLDVIMNVF---
OsPi-km2      : KWITSITTLNKLTISVTVLRTET--LEILHILPSLFSLTFAFSLSAAK------QDQDIIKDILENNKLDSDGEIVI------PAEGFKSLKLLRFFAPLVPKLSFLDK---
OsPi-b        : EWIKELQHLVKLKLVSTRLLEHDVAMEFLGELPKVEILVISPFKSEE-------IHFKPPQTGTAFVSLRVLKLAGL------WGIKSVKFEEGTMPKLERLQVQGRIE---
OsPi-ta       : EGAMPSVQRLNLRFNANEFKQYD--SKETGLEHLVALAEISARIGG--------TDDDESNKTEVESALR-------------TAIRKHPTPSTLMVDIQWVDWIFGAE---
OsPi36        : -------CLSHLDVKVQVVKEQD--METLGRLPMLCYLKLDSQYTRLISIKK-PADDGYFQKLRFFKTPRSFVRFDL------HGCESSSGASSFMPRLEHIEFSVDVR---
OsPi-d3       : HFVSVSNLNNLTFLRLAGSRIDENAFLNLEGLQQLVKLQLYDAYDG----------MNIYFHENSFPKLRILKIWGA------PHLNEIKMTKGAVASLTDLKFLLCPN---
OsPi-km1      : KMIKDHFRVLSCLDIRLCHKLEDDDQKFLAEMPNLQTLVLRFEALP---------RQPITINGTGFQMLESFR--VD------SRVPRIAFHEDAMPNLKLLEFKFYAGP--
OsBph14       : ELTLSDMTNFETWWDTNEVQGEELMFPEVEKLSIESCHRLTALPKASNAISE-SSGEVSTVCRSAFPALKEMKLYDLRIFQKWEAVDGTPREEATFPQLDKLEIRQCPELTT
OsXa1         : KWQILPLERLGLLVKLVLIKMRNATELSIPSLEELVLIALPSLNTCSCTSIRNLNSSLKVLKIKNCPVLKVFPLFEI------SQKFEIERTSSWLPHLSKLTIYNCPLS--
                                                                                    l
```

```
                 1360         *      1380         *      1400         *      1420         *      1440         *      1460
NRTP1    : DCGDATRSRVE--------------------------AAEASVRNAASAHPGCPRIEMINRYSKTS----------------------------------------------
NSL1     : DCNGSTGGELE--------------------------AAKASIKSSVNKLPGQPKLNLSTLNENMLVHEEAN----------------------------------------
OsPi-km2 : EVHLKVSDGAE--------------------------AITKFLVNDLKDNTEKPKVFVDGIVTA------------------------------------------------
OsPi-b   : RAARAAGADYE--------------------------TAWEEEVQEARRKGGELKRKIREQLARNPNQPIIT----------------------------------------
OsPi-ta  : NLQGLLSFFLS--------------------------LPWLLSLPAMHLQPDLMIV--------------------------------------------------------
OsPi36   : HCKDALAAEVQ--------------------------EAEVALANAAHGHPNRPVLRTDRAYERMMRSPDDDKPT------------SWSDQKVFEIHVYPSSNDHHRYLSY
OsPi-d3  : TAEELVDRIRQ--------------------------KKERMICDVQRVYVGFIRNGVLAAERIQ-----------------------------------------------
OsPi-km1 : TIDVVKKEAEE--------------------------HPNRPITLLINAGYKEISTESHGSSENIAGSSGIDTEP------------AQAQHDNLPAVRDDYKGKGILLDGR
OsBph14  : STDDTETASVAKQQD----------------------SSDLVIEDEKWSHKSPLELMVLSRCNLLFSHPSALALWTCFAQLLDLKIRYVDALVSWPEEVFQGLVSLRKLEIS
OsXa1    : YSQETLQPCFSGNLTLLRKLHVLGNSNLVSLQLHSCTALEELIIQSCESLSSLDGLQLLGNLRLLRAHRCLSGHGEDGRCILPQSLEELYIHEYSQETLQPCFSGNLTLLRK

                    *      1520         *      1540         *      1560         *      1580         *      1600         *
NRTP1    : ----------------------------------------------------------------------------------------------------------------
NSL1     : ----------------------------------------------------------------------------------------------------------------
OsPi-km2 : ----------------------------------------------------------------------------------------------------------------
OsPi-b   : ----------------------------------------------------------------------------------------------------------------
OsPi-ta  : ----------------------------------------------------------------------------------------------------------------
OsPi36   : DHHINHPTLEINRMEESISDQHQQVPENN-----------------------------------------------------------------------------------
OsPi-d3  : ----------------------------------------------------------------------------------------------------------------
OsPi-km1 : ----------------------------------------------------------------------------------------------------------------
OsBph14  : EVPNLPASLKLLEIRGCPGLESIVFNQQQDRTMLVSAESFAEQDKSSLISGSTSETNDHVLPRLESLVINWCDRLEVLHLPPSIKKLGIYSCEKLRSLSVKLDAVRELSIRH
OsXa1    : LLGNLRLLRAHRCLSGHGEDGRCILPQSLEELYIHEYSQETLQPCFSGNLTLLRKLHVLGNSNFVSLQLHSCTALEELIIQSCESLSSLDGLQLLGNLRLLQAHRCLSGHGE

                 1660         *      1680         *      1700         *      1720         *      1740         *      1760
NRTP1    : ----------------------------------------------------------------------------------------------------------------
NSL1     : ----------------------------------------------------------------------------------------------------------------
OsPi-km2 : ----------------------------------------------------------------------------------------------------------------
OsPi-b   : ----------------------------------------------------------------------------------------------------------------
OsPi-ta  : ----------------------------------------------------------------------------------------------------------------
OsPi36   : ----------------------------------------------------------------------------------------------------------------
OsPi-d3  : ----------------------------------------------------------------------------------------------------------------
OsPi-km1 : ----------------------------------------------------------------------------------------------------------------
OsBph14  : PQAYSSLTSLEIRGCSGIKVLPPSLQQRLDDIEDKELDACYEEAEAEPKSRHRQSAISRLMCLK------------------------------------------------
OsXa1    : VLGNSNLVSLQLHSCTALEELIIQSCESLSSLDGLQLLGNLRLLQAHRCLSGHGEDGRCILPQSLEELYIHEYSQETLQPCFSGNLTLLRKLHVLGNSNLVSLQLHSCTSLE
```

Figure S2. Amino acid alignment of NRTP1 with other CC-NB-LRR proteins identified in rice. Substitution from D to G in NRTP1 is marked by a red arrow. Substitutions in NSL1 are marked in red.
