# Supplementary material for: A semi-dominant mutation in a CC-NB-LRR-type protein leads to a short-root phenotype in rice
Source: Rice (N Y). 2018 Oct 3;11:54. doi: 10.1186/s12284-018-0250-1 (PMC6170248; doi:10.1186/s12284-018-0250-1)
Supplement: Supplementary file 5 — Figure S3. dCAPS marker for molecular identification. (A) DNA sequences of wild type and mutant. The mutated base is marked in red. The dis-matched base to the primer 17410-L is marked by red fork. (B) PCR products of wild type and mutant sequences. A point mutation is introduced after the PCR amplification (marked in green), resulting an Eco91I recognition site in mutant. (PDF 62 kb) [file 12284_2018_250_MOESM5_ESM.pdf]

Figure S3

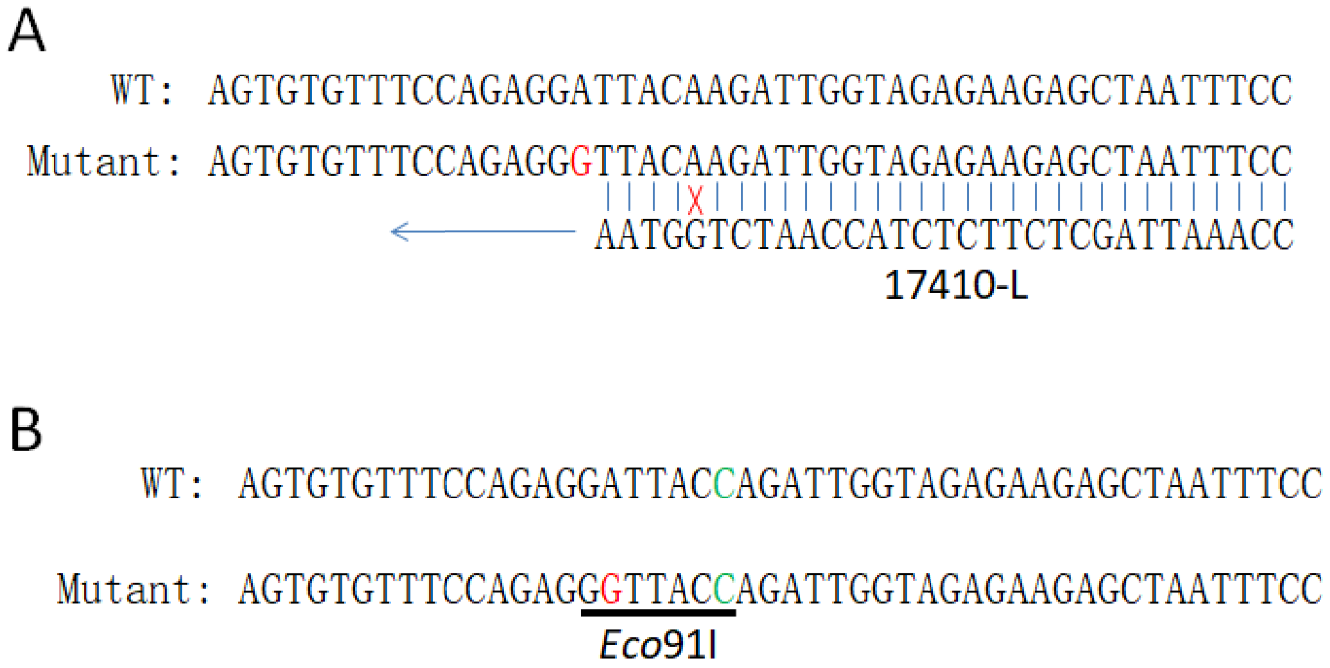

Figure S3. dCAPS marker for molecular identification. (A) DNA sequences of wild type and mutant. The mutated base is marked in red. The dis-matched base to the primer 17410-L is marked by red fork. **(B)** PCR products of wild type and mutant sequences. A point mutation is introduced after the PCR amplification (marked in green), resulting an *Eco*91I recognition site in mutant.
