# Supplementary material for: A semi-dominant mutation in a CC-NB-LRR-type protein leads to a short-root phenotype in rice
Source: Rice (N Y). 2018 Oct 3;11:54. doi: 10.1186/s12284-018-0250-1 (PMC6170248; doi:10.1186/s12284-018-0250-1)
Supplement: Supplementary file 6 — Figure S4. Co-segregation of phenotype and genotype. All the homozygous mutants produced higher mobility bands than the wild type. All the heterozygous mutants produced double bands. Bar = 2 cm. (PDF 2864 kb) [file 12284_2018_250_MOESM6_ESM.pdf]

Figure S4

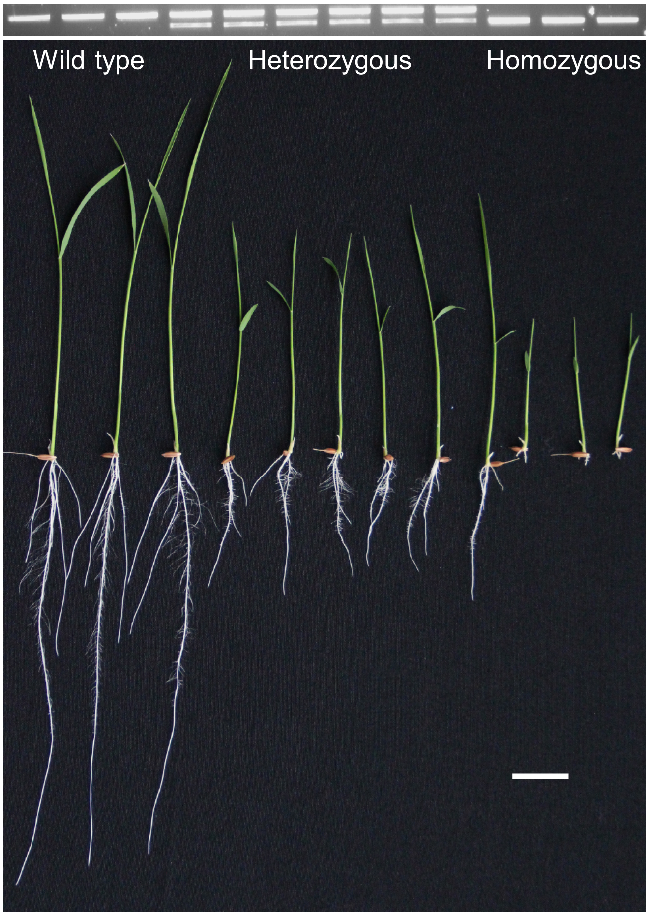

Figure S4. Co-segregation of phenotype and genotype. All the homozygous mutants produced higher mobility bands than the wild type. All the heterozygous mutants produced double bands. Bar = 2 cm.
