# Supplementary material for: A semi-dominant mutation in a CC-NB-LRR-type protein leads to a short-root phenotype in rice
Source: Rice (N Y). 2018 Oct 3;11:54. doi: 10.1186/s12284-018-0250-1 (PMC6170248; doi:10.1186/s12284-018-0250-1)
Supplement: Supplementary file 7 — Figure S5. RT-PCR analysis of NRTP1 expression after SA and JA treatments. Untreated roots are positive control. Numbers on the right are cycles in PCR. (PDF 166 kb) [file 12284_2018_250_MOESM7_ESM.pdf]

Figure S5

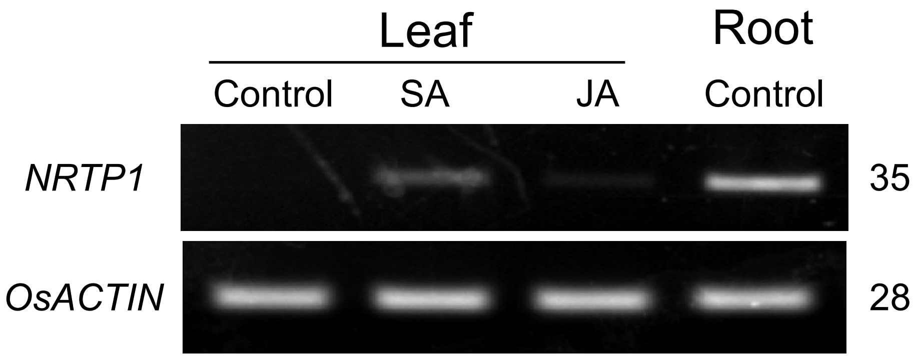

Figure S5. RT-PCR analysis of *NRTP1* expression after SA and JA treatments. Untreated roots are positive control. Numbers on the right are cycles in PCR.
