# Supplementary material for: A semi-dominant mutation in a CC-NB-LRR-type protein leads to a short-root phenotype in rice
Source: Rice (N Y). 2018 Oct 3;11:54. doi: 10.1186/s12284-018-0250-1 (PMC6170248; doi:10.1186/s12284-018-0250-1)
Supplement: Supplementary file 8 — Figure S6. Phenotypes of transgenic plants overexpressing NRTP1. (A) Comparison of phenotypes between wild type (WT) and transgenic plants overexpressing NRTP1. Line 1 and line 2 indicate independent transgenic lines. RT-PCR confirmation is shown at the bottom of the figure; the first row is the expression of NRTP1, the second row is the expression of the OsActin control. (PDF 1354 kb) [file 12284_2018_250_MOESM8_ESM.pdf]

Figure S6

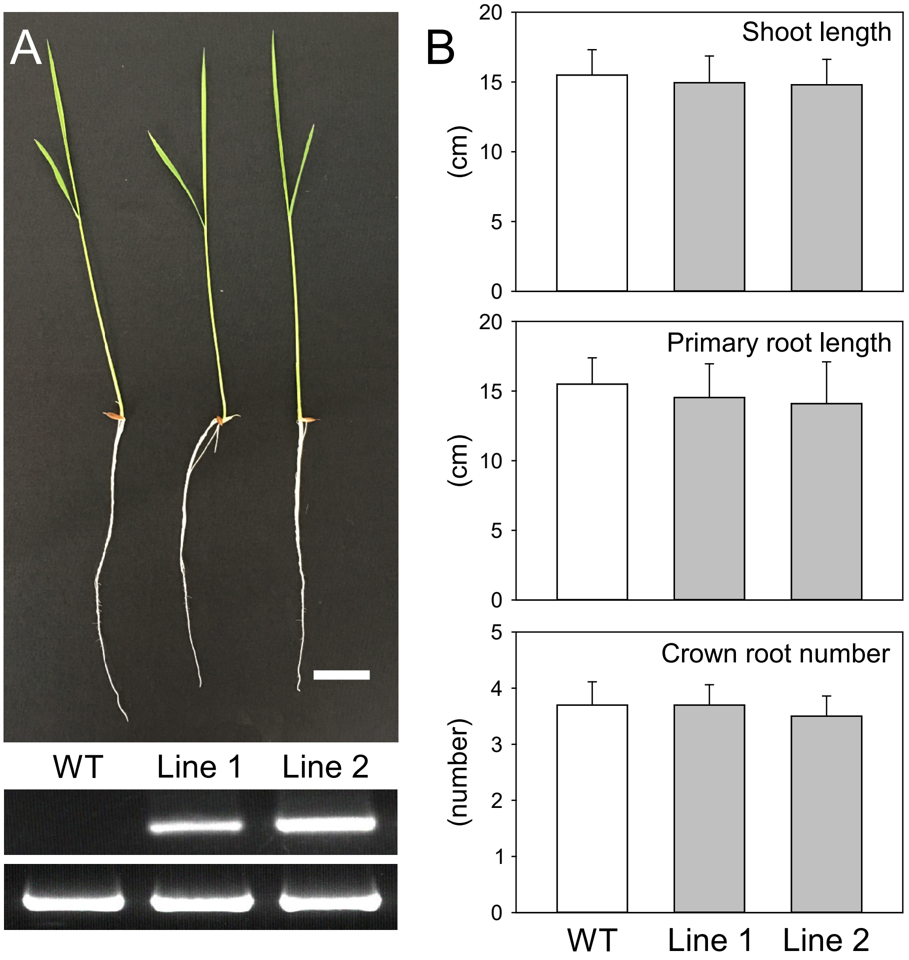

Figure S6. Phenotypes of transgenic plants overexpressing *NRTP1*. (A) Comparison of phenotypes between wild type (WT) and transgenic plants overexpressing *NRTP1*. Line 1 and line 2 indicate independent transgenic lines. RT-PCR confirmation is shown at the bottom of the figure; the first row is the expression of *NRTP1*, the second row is the expression of the *OsActin* control.
