# Supplementary material for: A semi-dominant mutation in a CC-NB-LRR-type protein leads to a short-root phenotype in rice
Source: Rice (N Y). 2018 Oct 3;11:54. doi: 10.1186/s12284-018-0250-1 (PMC6170248; doi:10.1186/s12284-018-0250-1)
Supplement: Supplementary file 12 — Figure S7. KEGG enrichment of differentially expressed genes. (PDF 40 kb) [file 12284_2018_250_MOESM12_ESM.pdf]

Figure S7

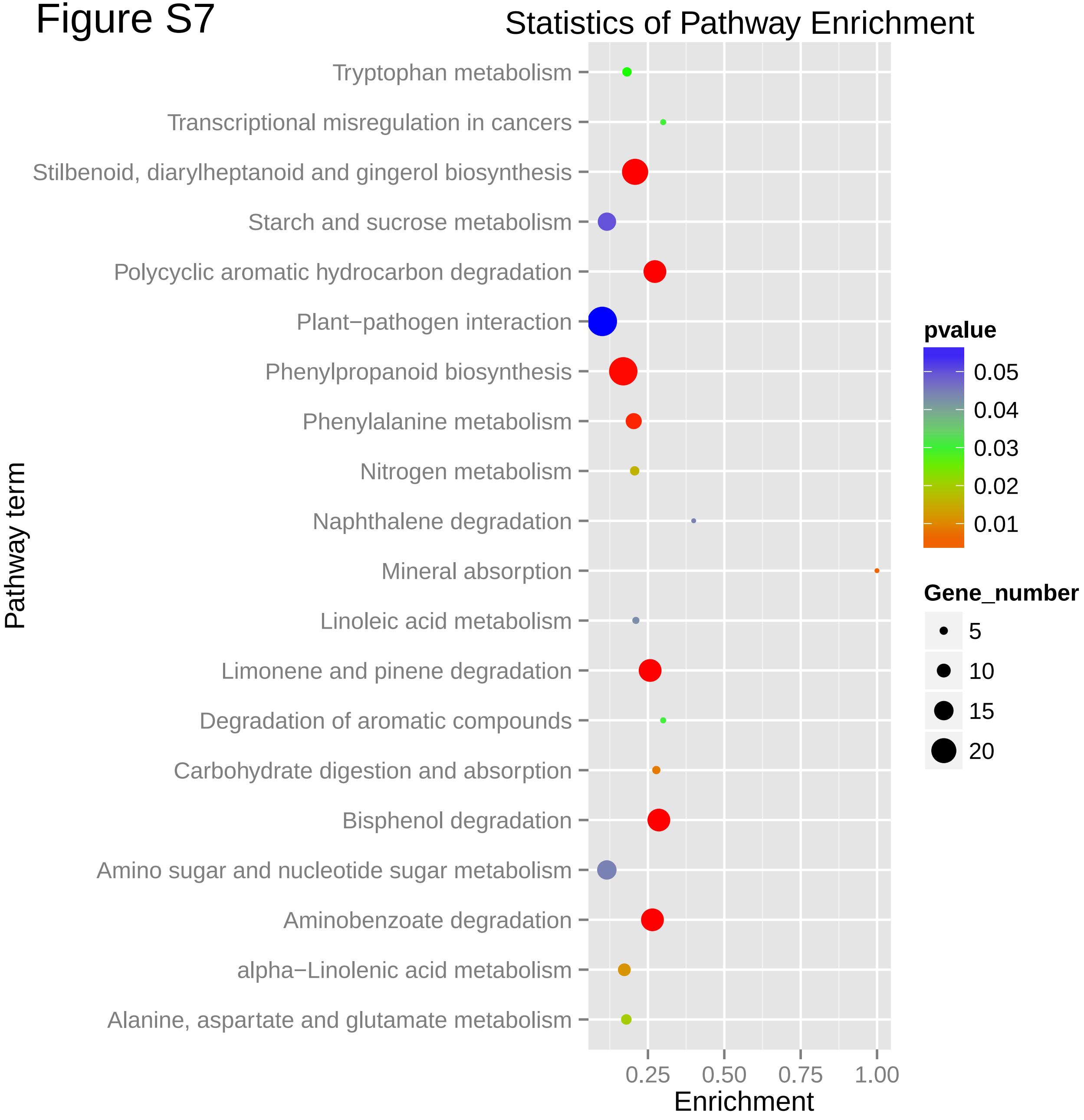

Figure S7. KEGG enrichment of differentially expressed genes.
