# Supplementary material for: A semi-dominant mutation in a CC-NB-LRR-type protein leads to a short-root phenotype in rice
Source: Rice (N Y). 2018 Oct 3;11:54. doi: 10.1186/s12284-018-0250-1 (PMC6170248; doi:10.1186/s12284-018-0250-1)
Supplement: Supplementary file 14 — Figure S8. Down-regulated and up-regulated genes in the most significantly enriched pathways in nrtp1-D. (PDF 79 kb) [file 12284_2018_250_MOESM14_ESM.pdf]

Figure S8

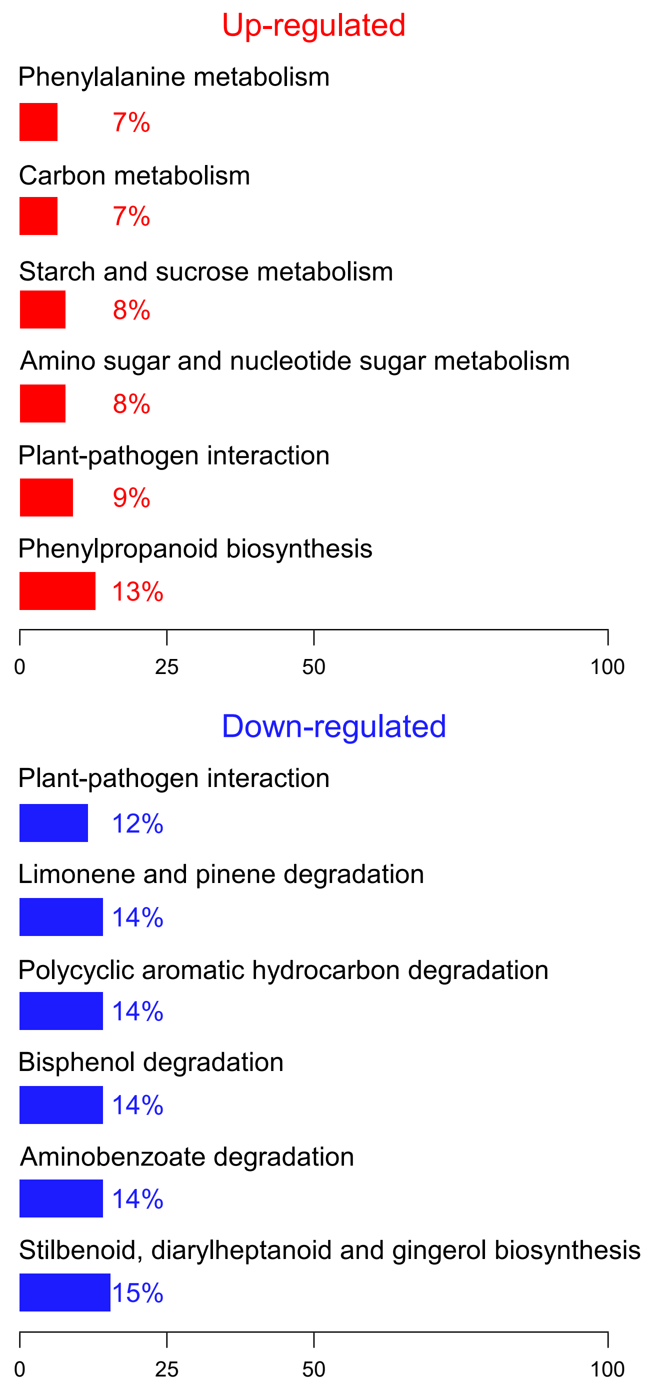

Figure S8. Down-regulated and up-regulated genes in the most significantly enriched pathways in *nrtp1-D*.
