# Supplementary material for: A semi-dominant mutation in a CC-NB-LRR-type protein leads to a short-root phenotype in rice
Source: Rice (N Y). 2018 Oct 3;11:54. doi: 10.1186/s12284-018-0250-1 (PMC6170248; doi:10.1186/s12284-018-0250-1)
Supplement: Supplementary file 16 — Figure S9. NO and H2O2 contents in leaves and root tips of 7-d-old wild type and homozygous nrtp1-D. Asterisks indicate significant differences (P < 0.01; Student’s t-test). FW, fresh weight. Data of independent experiments are shown (mean ± SD; n = 3). (PDF 183 kb) [file 12284_2018_250_MOESM16_ESM.pdf]

Figure S9

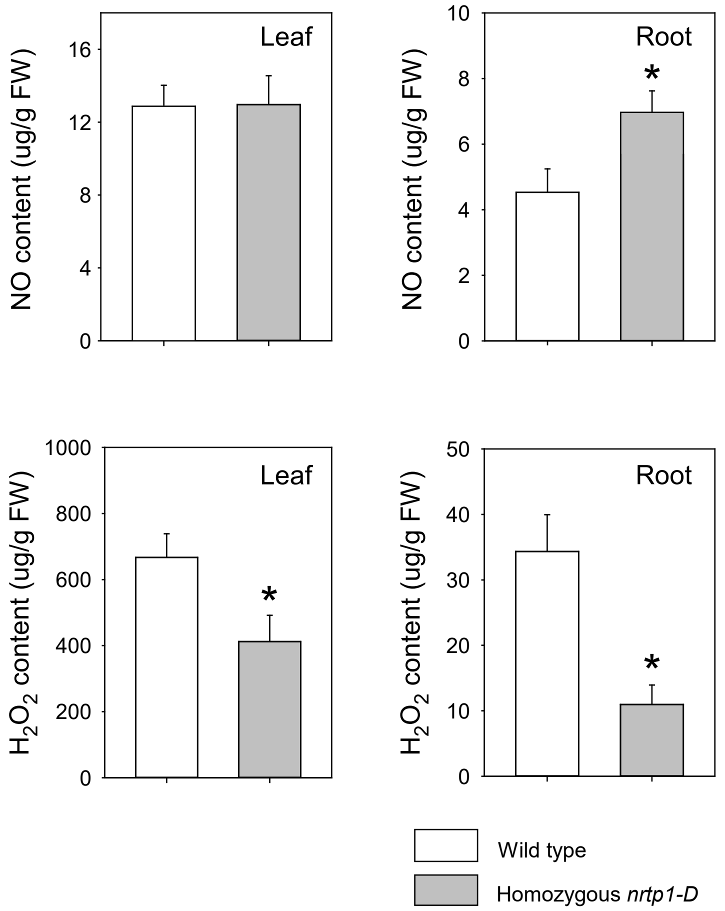

Figure S9. NO and H_2O_2 contents in leaves and root tips of 7-d-old wild type and homozygous *nrtp1-D*. Asterisks indicate significant differences ($P < 0.01$; Student's *t*-test). FW, fresh weight. Data of independent experiments are shown (mean ± SD; $n = 3$).
